# Enhanced blood-brain-barrier penetrability and tumor-targeting efficiency by peptide-functionalized poly(amidoamine) dendrimer for the therapy of gliomas

**DOI:** 10.7150/ntno.38954

**Published:** 2019-09-19

**Authors:** Changliang Liu, Zijian Zhao, Houqian Gao, Iman Rostami, Qing You, Xinru Jia, Chen Wang, Ling Zhu, Yanlian Yang

**Affiliations:** 1 CAS Key Laboratory of Standardization and Measurement for Nanotechnology, CAS Key Laboratory of Biological Effects of Nanomaterials and Nanosafety, CAS Center for Excellence in Nanoscience, National Center for Nanoscience and Technology, Beijing 100190, China; 2 University of Chinese Academy of Sciences, Beijing 100049, China; 3 Department of Chemistry, Peking University, Beijing 100871, China

**Keywords:** poly(amidoamine) dendrimer, central nervous system, dual-targeting, blood-brain-barrier, glioblastoma

## Abstract

Glioblastoma is one of the most common primary tumor types of central nervous system (CNS) with high malignance and lethality. Although many treatment options are currently available, the therapy of brain cancers remains challenging because of blood-brain-barrier (BBB) which prevents most of the chemotherapeutics into the CNS. In this work, a poly(amidoamine) dendrimer-based carrier was fabricated and modified with angiopep-2 (Ang2) peptide that has been demonstrated to bind to low density lipoprotein receptor-relative protein-1 (LRP1) on the endothelial cells of BBB and could therefore induce BBB penetration of the carrier. To improve tumor-targeting effect towards the glioma sites, the dendrimer was simultaneously functionalized with an epidermal growth factor receptor (EGFR)-targeting peptide (EP-1) which was screened from a “one-bead one-compound” (OBOC) combinatorial library. EP-1 peptide was demonstrated to have high affinity and specificity to EGFR at both the molecular and cellular levels. The dual-targeting dendrimer exhibited outstanding BBB penetrability and glioma targeting efficiency both *in vitro* and *in vivo*, which strikingly enhanced the anti-gliomas effect of the drugs and prolonged the survival of gliomas-bearing mice. These results show the potential of the dual-targeting dendrimer-based carrier in the therapy of gliomas through enhancing BBB penetrability and tumor targeting.

## Introduction

Glioblastoma multiforme (GBM) is one of the most common primary tumor types in central nervous system (CNS) with high malignance, poor prognosis and high mortality. Statistically, over 30% of CNS associated tumors and 80% of aggressive brain cancers are gliomas. Although great progresses have been made in the treatment of gliomas, the recurrence and mortality of gliomas are still high 1-4. The main bottleneck for the treatment of gliomas is blood-brain-barrier (BBB). BBB is mainly formed depending on the complex tight junctions between the adjacent endothelial cells and the other constituents, including extracellular matrix, astrocytes and pericytes, which can prevent diffusion of the toxic foreign substances into the brain parenchyma. However, it also prevents the penetration of therapeutic drugs such as the drugs against gliomas, Alzheimer's disease (AD), or Parkinson's disease (PD) into the CNS 5-8. In general, BBB could block 98% small molecule drugs and almost all of the large molecule drugs. Only a small number of hydrophobic drugs with the molecular weight less than 500 Da could pass through the BBB, such as temozolomide 9-11. Although temozolomide could cross the BBB, and is commonly used as a standard chemotherapeutic for GMB, the therapeutic efficacy of temozolomide is still unsatisfactory due to the limited accumulations of drugs in the brain and the low targeting efficiency in glioma sites. Besides, the diffusion of chemotherapeutics in normal tissues due to the short-circulation time and non-specific accumulation *in vivo* would also bring harmful side effect and even fatal damage 12, 13.

To meet the requirement of brain cancer therapy, great efforts have been made to improve the BBB penetrability and targeting effect of the therapeutics. Modifying the drugs with functional moieties is one feasible strategy. For example, attaching charged or lipophilic groups on the pharmaceutical molecule could improve the adsorption of drugs onto the endothelial cells of BBB, which would enhance the BBB penetrability by adsorption-mediated transcytosis 14-16. Modification of PEG chain on the pharmaceutical molecules could also improve the BBB penetrability by prolonging the circulation time *in vivo* which would increase the probability of the interactions between the drugs and BBB 17. Additionally, drugs could target endothelial cells of BBB and traverse into the brain by receptor-mediated transcytosis or transporter-mediated transcytosis after being modified with peptide ligands or antibodies 18-20. This strategy has attracted much attention, yet the efficacy of drugs and the pharmacokinetics remain to be confirmed. With the development of nanotechnology and nanomedicine in the recent decades, various drug delivery vehicles, such as liposomes 21-23, hydrogels 24, micelles 25, 26, polymers 27-29 and inorganic nanoparticles 30-35 have been developed to incorporate therapeutics and to deliver them into tumor sites and CNS.

Delivering drugs through the targeting endogenous transport system of BBB is a highly selective and non-invasive delivery route for the CNS disease 36, 37. Therefore, nanocarriers were modified with various BBB-penetrating ligands to improve the receptor-mediated transcytosis and transporter-mediated transcytosis, which could enhance the ability of drug carriers across the BBB and deliver the therapeutic reagents into lesions without disrupting homeostasis and biological barriers 5. Previous researches have determined several receptors and transporters for BBB penetrating by transcytosis, including low-density lipoprotein receptor-relative protein-1 (LRP1) 38, 39, insulin receptor 40, 41, transferrin receptor (TfR) 42, 43, nicotinic acetylcholine receptor and choline transporter 44, 45 and a short peptide (RVG29) derived from rabies virus 29, 33. Among these receptors, LRP1, a large multi-ligand endocytic receptor belonging to the low-density lipoprotein receptor family, has attracted wide interest in the therapy and diagnose of CNS disease. As a ligand of the LRP1, angiopep-2 peptide (Ang2, TFFYGGSRGKRNNFKTEEY), which was derived from the Kunitz domains of aprotinin and other human proteins, has been proven to specifically bind with LRP1 and promote drug carriers entering into the brain by LRP1-mediated transcytosis 19, 46. Notably, angiopep-2 peptide modified PTX has been in clinical trial for the therapy of breast cancer brain metastasis 20. Therefore, we chose Ang2 peptide as a BBB-penetrating ligand to enhance the receptor-mediated transcytosis of drug delivery system for glioma therapy in this work after comprehensive consideration.

Dendritic polymer, such as poly(amidoamine) (PAMAM) dendrimer, are considered as one of the most promising polymer architectures for drugs and gene targeting delivery in cancer therapy. Compared with other polymer carriers such as liposome and micelle, dendrimer-based carriers have many advantages. First, dendrimers are hyperbranched macromolecules synthesized through chemosynthesis, instead of self-assembly through noncovalently interaction, which makes them stable in the complicated blood circle system. Besides, PAMAM dendrimer-based carriers could be modified by various ligands and encapsulate many chemotherapeutics due to their abundant periphery amino groups and interior cavity structures 47. In addition, the precise size, structure and molecular weight of the dendrimers are similar to some important proteins and bio-structures, such as insulin, cytochrome and hemoglobin, which make the dendrimers widely used in many fields, such as immunodiagnostic, gene delivery and drug encapsulation as preclinical carriers 48, 49. In the last decades, several PAMAM dendrimer-based drug delivery systems have been developed to explore their potential usage for brain cancer therapy 28, 47. All the studies demonstrated that the functionalized dendrimer-based nanoparticles were considerable drug delivery carriers in the targeting therapy of brain tumor which could transport across the BBB through receptor-mediated transcytosis and then target and accumulate in the glioma site with assistance of targeting ligands.

**Scheme 1:**
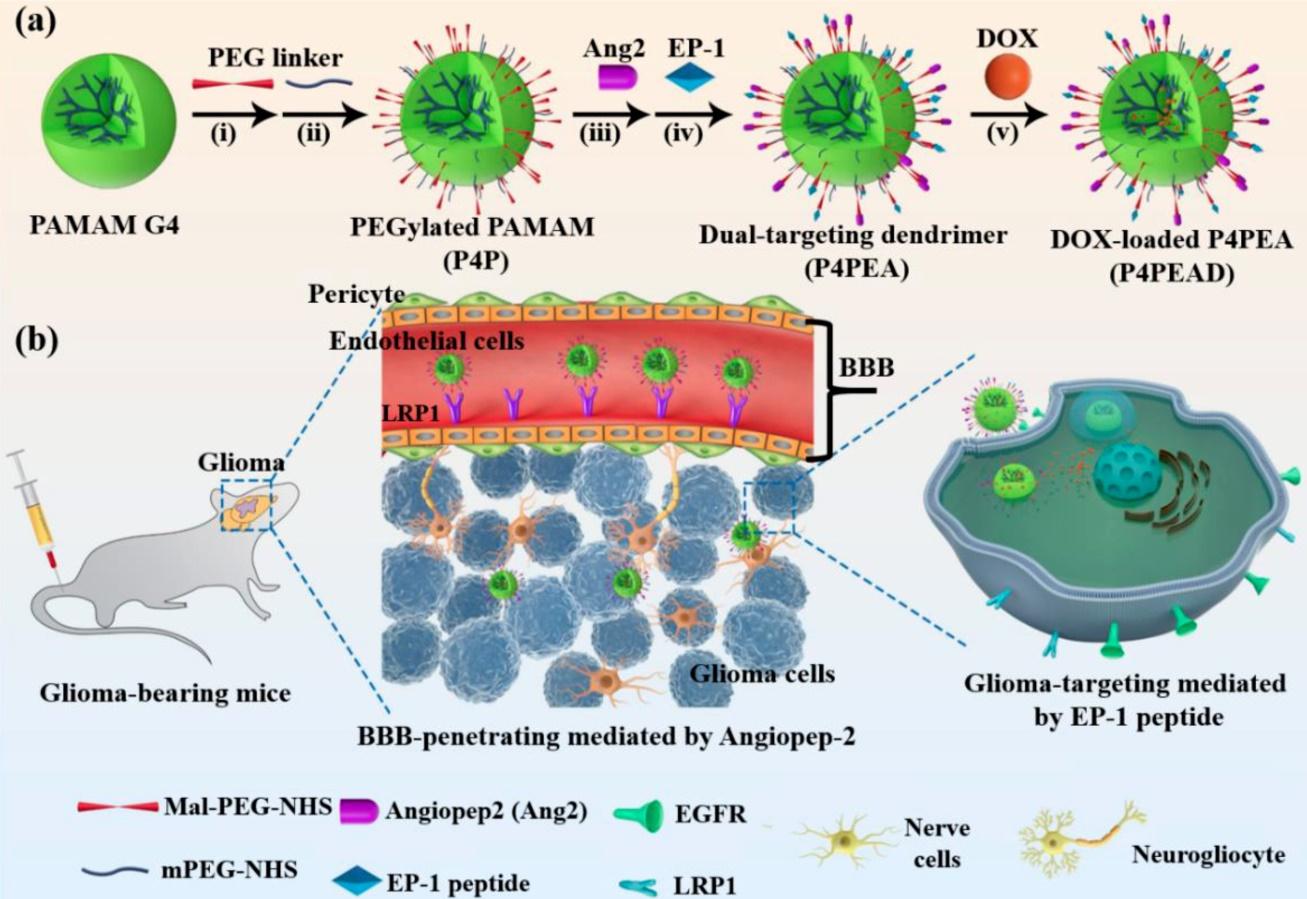
(a) Construction of the dual-targeting dendrimer-based drug carrier. Reagents and conditions: (i) H_2_O, room temperature (RT), 30 min; (ii) H_2_O, RT, 30 min; (iii) H_2_O, RT, Argon, 1 h; (iv) H_2_O, RT, Argon, 4 h; (v) stirring in dark place, 24 h. (b) Schematic illustration of glioma targeting therapy across the BBB using the dual-targeting drug delivery system.

In this present work, a BBB-penetrating drug carrier was designed and fabricated based on the fourth generation PAMAM dendrimer (P4) by conjugating Ang2 peptide. To improve the gliomas targeting effect after the carrier crossing BBB, another peptide targeting epidermal growth factor receptor (EGFR) was screened through a “one-bead one-compound” (OBOC) combinatory library and conjugated on the dendrimer simultaneously as increased gene amplification and protein overexpression of EGFR has been found in almost 50% of glioma patients 3. Afterwards, the anti-tumor drug, doxorubicin (DOX), was incorporated into the interior cavities by non-covalent interactions (**Scheme 1**). The encapsulated DOX could controllably release from the dendrimer responding to the weak acidic pathological environment, which reduced toxics for normal cells and tissues both *in vitro* and *in vivo*. Meanwhile, modification of the dendrimer carriers with the peptides significantly improved the BBB penetrability of DOX in a BBB model *in vitro* and enhanced its anti-glioma effect after traversing the BBB. *In vivo* experiments also demonstrated that the dual-targeting modification of the dendrimer strikingly improved the BBB penetrability and glioma targeting effect of the drug delivery system, which improved the anti-glioma efficacy of DOX and prolonged the survival of the glioma-bearing mice.

## 2. Materials and Methods

### 2.1 Materials

Fourth generation PAMAM dendrimer with an ethylenediamine core (10 wt% in methanol, MW 14215), transferrin (Tf), human serum albumin (HSA) and O-[(N-succinimidyl) succinyl-aminoethyl]-O'-methyl polyethylene glycol (mPEG-NHS, MW 750) were purchased from Sigma-Aldrich (St. Louis, MO, USA). Maleimide PEG Succinimidyl Carboxymethyl Ester (Mal-PEG-NHS, MW 2000) was purchased from JenKem technology Co., Ltd. (Beijing, China). Angiopep-2 (Ang2, TFFYGGSRG KRNNFKTEEYC, MW 2404) peptide was synthesized by Guoping Pharmaceutical Co., Ltd. (Anhui, China). Doxorubicin hydrochloride was purchased from Meilun Biotechnology Co., Ltd. (Dalian, China). Minimum Essential Medium (MEM), Dulbecco's Modified Eagle Medium (DMEM) and penicillin/streptomycin were purchased from Gibco (ThermoFisher Scientific, USA). Endothelial Cell Medium (ECM) was purchased from Sciencell (USA). Plexera Nanocapture bare golds chip (thickness: 47.5 nm, size: 2.5 × 7.5 cm) was purchased from Plexera (USA). EGFR, HER2, epidermal growth factor (EGF) and fibroblast growth factor (FGF) were purchased from Sino Biological Inc. (Beijing, China). IgM and IgG were purchased from abcam (Shanghai, China). 9-Fluorenylmethoxy-carbonyl protected amino acids were purchased from GL Biochem (Shanghai, China). 2-(1H-benzotriazole-1-yl)-1, 1, 3, 3-tetramethyluoronium hexafluorophosphate (HBTU), trifluoroacetic acid (TFA), triisopropylsilane (Tips) and streptavidin (SA) coated magnetic beads (dimer: 1 μm) were purchased from Sigma-Aldrich. N-methylmorpholine (NMM), N, N'-dimethylforma-mide (DMF) and piperidine were purchased from Beijing Chemical plant (China). Biotin labeling kit was purchased from Solulink (USA). Teflon microchannel and bi-functional microchip were kindly offered from Prof. Zhiyuan Hu. All regents were of analytical grade and used without any purification. Deionized water used in all experiment was obtained from Milli-Q Integral 3 (Merck Millipore. France).

### 2.2 Cell lines and animals

Human glioblastoma U87-MG cells was purchased from Cell Bank of Chinese Academy of Science (Shanghai, China). Human breast cancer MDA-MB-231 and MCF-7 cells were purchased from Chinese Academy of Medical Science & Peking Union Medical College (Beijing, China). Human brain microvascular endothelial HBMEC cells was purchased from iCell Bioscience Inc. (Shanghai, China). U87-MG cells were cultured in MEM medium supplemented with 10% fetal bovine serum (FBS), 1% penicillin/streptomycin, 1.5 g/L sodium carbonate and 0.11 g/L sodium pyruvate. MDA-MB-231 and MCF-7 cells were cultured in DMEM medium supplemented with 10% FBS and 1% penicillin/streptomycin. HBMEC cells were cultured in ECM medium supplemented with 5% FBS, 1% endothelial cell growth supplements (ECGS) and 1% penicillin/streptomycin. All the cells were cultured at 37 ℃ with 5% CO_2_.

Female BALB/c nude mice (6-8 weeks, 18-20 g) and female CB-17 SCID mice (6-8 weeks, 16-18g) were purchased from Charles River Laboratory Animal Technology Co., Ltd. (Beijing, China). All animal procedures were approved by the Animal Ethics Committee of National Center for Nanoscience and Technology, and performed in strict accordance with the Guidelines for Care and Use of Laboratory Animals of National Center for Nanoscience and Technology, Chinese Academy of Sciences.

### 2.3 Detection of EGFR and LRP1 expression in cell lines

EGFR expression in MDA-MB-231, MCF-7, U87-MG and HBMEC cells was evaluated by flow cytometry. Briefly, 5 × 10^6^ cells were harvested and incubated with primary antibody of EGFR (ab30, abcam) for 1 h, followed with Alexa Fluor® 647 labeled rabbit/anti-mouse IgG (4410, CST) for 30 min. Then the cells were washed by PBS for three time. Finally, the cells were resuspended into 200 μL PBS and analyzed using BD Accuri^TM^ C6 system (BD, USA). By contrast, nontreated cells and non-specific anti-mouse IgG (5415, CST) treated cells were used as blank control and isotype control, respectively.

EGFR expression in MDA-MB-231, MCF-7, U87-MG and HBMEC cells was further detected by confocal imaging. Cells were seeded in glass bottom dishes with the density of 5 × 10^4^ cells per dish. After incubating for 24 h, the cells were fixed by 4% paraformaldehyde. Then the cells were incubated with primary antibody of EGFR (ab30, abcam) for 1 h and followed with Alexa Fluor® 647 labeled rabbit/anti-mouse IgG (4410, CST) for 30 min at room temperature. Finally, the nuclei were stained by Hoechst and the cells were analyzed using laser scanning confocal microscope (LSCM, Zeiss 710, Germany) at the channel of 405 nm and 633 nm.

LRP1 expression in U87-MG and HBMEC cells was performed by the same procedure. The primary antibody of LRP1 (ab92544, abcam) and Alexa Fluor® 488 labeled coat/anti-rabbit IgG (4412, CST) were used in this experiment.

### 2.4 Synthesis of the “one-bead one-compound” (OBOC) combinatorial library towards EGFR

The OBOC combinatorial library comprised of X_1_X_2_X_3_X_4_X_5_X_6_X_7_X_8_X_9_GM was synthesized by solid phase peptide synthesis (SPDP) using Tentagel beads as solid supports according to the previous report 50, 51. X_1_ represent either F, W, R or K residues. X_2_ represent either V, D, K or E residues. X_3_ represent either K, F, L or R residues. X_4_ represent either E, V, R or Y residues. X_5_ represent either L, E, F or Y residues. X_6_ represent either Y, K, D or V residues. X_7_ represent either E, L, W or R residues. X_8_ represent either E, R, F or D residues. X_9_ represent either Y, K, V or L residues. The methionine residue in C-terminal was designed as the cleavage site of cyanogen bromide. The whole synthesis processes were shown in** Scheme S**1: In the coupling step, the beads were split equally according to the mutation of amino acids, then equivalent of HBTU and Fmoc-amino acids were dissolved in 0.4 mol/L NMM solution and added into the beads, the mixture was allowed to react for 60 min at room temperature. In the deprotection step, the beads were pooled together and 20% (v/v) piperidine was added to remove the Fmoc group by reacting for 20 min. All the synthesis process was carried out in dehydrated DMF. After elongation, cleavage reagent consisted of 95% (v/v) TFA, 2.5% (v/v) deionized water and 2.5% (v/v) Tips was introduced into the vessel to cleave the side chain protecting groups of each residue for 2 h.

### 2.5 Sorting and identification of the positive peptide beads

EGFR was biotinylated using ChromaLink Biotin Protein Labelling Kit (Catalog #B-9007-105K) before the experiment. Peptide beads were incubated with 5% skimmed milk to block the nonspecific sites. Afterwards, the peptide beads were incubated with the biotinylated EGFR for several hours at 4 ℃. Then streptavidin (SA) coated magnetic beads were added into the mixture to label positive beads. After incubating for 1 h, all the peptide beads were collected by centrifugation and flowed through a Teflon microchannel (diameter: 1 mm, flow rate: 600 μL/min) with a magnet closely next to the outer wall of the tube. The trapped positive beads by the magnet were put into a bio-functional microchip with one bead in one well and cleaved by hydrogen bromide overnight. Then the secondary mass spectrometry of the peptides was recorded by Matrix-assisted laser desorption/ionization time-of-flight mass spectrometry (MALDI-TOF MS) analysis on a Bruker ULTRAFLEXTREME mass spectrometer (Bruker Daltonics, Germany) which equipped with a nitrogen laser (wavelength = 337 nm, laser pulse duration = 3 ns) with reflectron and positive-ion modes. Finally, the peptide sequences would be obtained by analyzing the secondary mass spectrometry using Mascot software.

### 2.6 Surface plasmon resonance imaging (SPRi)

Affinity of peptides to EGFR was analyzed by SPRi on a PlexArray HT system (Plexera Bioscience, Seattle, WA, USA) according to a protocol previously reported 50. Briefly, the peptides were dissolved into deionized water with concentration of 10 mM. Then 1.5 μL of the peptide aqueous solution was dropped on the surface of the bare gold chip and incubated at 4 ℃ overnight in a humid box. Afterwards, the chip was washed with PBST (PBS buffer with 0.1 wt% tween20) and deionized water for 10 min, respectively. Then the peptide matrix coated gold chip was blocked by 5% (w/v) non-fat milk for 2 h before use. Then the gold chip was put into the PlexArray HT system for SPRi analysis according to the following cycle of injection: 1) washing the SPRi microchannel with PBST buffer at a constant rate of 2 μL/s to obtain a stable baseline; 2) EGFR protein was diluted with PBST to concentrations of 5.68, 11.4, 22.8, 45.6, 91.2 nmol/L, then the proteins were injected into the SPRi microchannel at 2 μL/s for binding; 3) washing the surface of the chip with PBST at 2 μL/s for 300 s; 4) regeneration with 0.5% (v/v) H_3_PO_4_ at 2 μL/s for 300 s. All measurements were performed at 4 ℃. Real-time binding signal were recorded and analyzed by Data Analysis Module (Plexera Bioscience, Seattle, WA, US) and the kinetic analysis was performed using BIAevaluation 4.1 software (Biacore, Inc.).

To determine the binding specificity of the peptides towards EGFR, several proteins, including EFGR, HER2, Tf, HSA, IgG, IgM, EGF and FGF flowed through the SPRi microchannel successively at a concentration of 45.6 nM following the same procedure mentioned above. The binding signal between the peptides and different proteins was recorded and analyzed by Data Analysis Module.

### 2.7 Detection of the binding affinity of EP-1 towards EGFR in cell lines

Affinity of the identified peptide towards EGFR in cell lines was detected by confocal imaging firstly. Approximately 5 × 10^4^ MDA-MB-231, U87-MG and MCF-7 cells were seeded into glass bottom dishes and cultured for 24 h at 37 ℃. Then FITC-labeled peptide was added into the cells at a final concentration of 40 μM and incubated for 1 h at 37 ℃. Then the medium was removed and the cells were washed with cold PBS for three times. Nuclei of the cells were stained with Hoechst before imaging. Confocal imaging was performed on a laser scanning confocal microscope (Zeiss 710, Germany). MDA-MB-231 and U87-MG cells with EGFR overexpression were used as positive terms and MCF-7 with low EGFR expression were used as negative control.

Meanwhile, affinity between the peptide and EGFR in cell lines was also tested by flow cytometry. U87-MG, MCF-7 and MDA-MB-231 cells were washed once with PBS and then resuspended with complete medium to 5 × 10^6^ cells/mL. 50 μL of the suspension was added into the tube and centrifuged at 800 g for 3 min and the supernatant was removed carefully. Then the FITC-labeled peptide was added into the cells at different concentrations in complete medium. After incubating for 1 h at room temperature, the cells were washed twice with PBS and suspended into 200 μL PBS. Finally, 1 × 10^4^ cells were analyzed by BD Accuri^TM^ C6 system (BD, USA).

### 2.8 Fabrication of the dual-targeting PAMAM dendrimer-based drug carrier

The dual-targeting drug delivery carrier was synthesized by conjugating the peptides on the surface of dendrimer according the previous report 47. The whole procedure was divided into two steps as shown in **Scheme 1a**. Firstly, PEGylated G4 PAMAM dendrimer (P4P) was prepared by reacting the surface amino groups of PAMAM dendrimer with both the PEGs bearing an NHS end group and the PEGs with NHS and Mal groups on both sides of the chain ends. Then the peptides were conjugated to the PEGylated PAMAM dendrimer through Michael addition reaction. Briefly, 5 mg (0.35 μmol) G4 PAMAM (P4) with 64 surface amino groups was dissolved in 5 mL deionized water, and then 17.5 mg (8.75 μmol) Mal-PEG-NHS were added into the dendrimer solution and allowed to stir gently for 30 min at room temperature. Then 6.6 mg (8.75 μmol) mPEG-NHS were added into the system to allow reacting for another 30 min. The resulting product was dialyzed against deionized water for 2 h to remove the residual PEG and the resulting solution was added into a clean flask. Afterwards, 6 mg (2.5 μmol) Ang2 peptide was added into the solution and the mixture was stirred under argon protection for 1 h at room temperature. Then 6 mg (4 μmol) EP-1 peptide was added and the mixture was allowed to react for another 4 h at the same condition. The resulting crude product was dialyzed against deionized water for 12 h to remove the residual peptides. The Ang2 and EP-1 peptides modified dendrimer was named PAMAM-PEG-EP1-Ang2 (P4PEA). The same procedure was used to synthesize the carriers modified with either Ang2 or EP-1 peptide (PAMAM-PEG-Ang2 (P4PA) and PAMAM-PEG-EP1 (P4PE).

### 2.9 Characterization of the dual-targeting dendrimer-based carrier

The chemical structure of the dual-targeting dendrimer was characterized by ^1^H NMR measurement on AVANCE III HD 400 MHz spectrometer (Bruker, USA) using D_2_O as solvent. ^1^H NMR (D_2_O, 400 MHz, ppm): δ 2.25-2.40 (-CH_2_*CH_2_*CONH-); δ 2.45-2.55 (-CH_2_*CH_2_*N<); δ 2.68-2.74 (-NCH_2_*CH_2_*CO-); δ 2.95-3.20 (-CONH*CH_2_*CH_2_- and -CH_2_*CH_2_*NH_2_); δ 3.32-3.75 (-*CH_2_CH_2_*O-); δ 6.79 (-CO*CH=CH*CO-); δ 3.30 (-O*CH_3_*). Size distribution and zeta potential of the dendrimer-based carriers were measured by Zetasizer Nano ZS nanoparticle size analyzer (Malvern Instruments Ltd., UK) at a concentration of 0.1 mg/mL in PBS. The samples were sonicated for 3 min and filtered with 200 nm filter membrane before detection. Morphological characterization of the dendrimer-based carriers was performed on a Ht-7700 transmission electron microscope (Hitachi, Japan) at acceleration voltage of 80 KV. TEM samples were made by dropping 2.5 μL samples (1 mg/mL) on carbon-coated grids and deposited for 30 min. Then the samples were negative stained by 1 wt% uranyl acetate for 25 s before test. Stability of the dual-functional dendrimer in water and PBS was detected by DLS within 72 h. Briefly, the samples were dissolved into deionized water and PBS at a concentration of 1 mg/mL. Then the particle size was detected by a Zetasizer Nano ZS nanoparticle size analyzer at 0 h, 12 h, 24 h, 48 h and 72 h.

### 2.10 Doxorubicin loading

Doxorubicin (DOX) was loaded into the interior of the dual-targeting dendrimer using an equilibrium dialysis method following the previous report 52.10 mg P4PEA was dissolved in 5 mL deionized water. 1 mg doxorubicin hydrochloride was dissolved in 1 mL methanol and neutralized with 3-fold molar excess of triethylamine. Then, the DOX solution was added into the P4PEA drop by drop with vigorous stirring and the mixture was allowed stirring in open air to evaporate the methanol solvent in dark. After stirring for 24 h in dark, the mixture solution was transferred to a dialysis bag (MWCO 8000) and dialyzed twice against deionized water under strict sink condition for 2 h to remove the free DOX. The encapsulation efficiency of DOX was determined by UV-visible scanning spectrophotometer (Lambda 950, Perkin Elmer Instruments Co. Ltd.) at 480 nm in deionized water. The loading of DOX for other carriers was performed with the same procedure. The DOX loaded different carrier forms were named P4PD, P4PED, P4PAD and P4PEAD, respectively.

### 2.11 *In vitro* release of DOX

*In vitro* release of the encapsulated DOX from P4PEA was studied using the dialysis bag method 53. A dialysis bag (MWCO 3500) containing 2 mL of P4PEAD aqueous solution at a DOX concentration of 200 μM was immersed in 45 mL PBS buffer (pH = 7.4 or 5.5) and incubated at 37 ℃. 1 mL buffer medium was taken out at each predetermined time intervals and the equal volume fresh buffer was added. The released DOX from the carrier was calculated with a standard curve draw by the fluorescent spectrometry with the excitation and emission wavelength of DOX at 480/595 nm. The release study was carried out for 24 h. All the tests were carried out in triplicate.

### 2.12 *In vitro* cytotoxicity assay

Cell cytotoxicity *in vitro* was measured by [3-(4, 5-dimetylthiazol-2-yl)-5-(3-carboxymethoxy-phenyl)-2-(4-sulfophenyl)-2H-tetrozolium, inner salt (MTS) assay in HBMEC and U87-MG cells. Briefly, cells were seeded into 96-well plates at a density of 4000 cells per well in 200 μL medium. After culturing for 24 h, various drug formulations were added at DOX concentration ranging from 0 to 31.25 μM. After incubating for another 48 h, the cell viability was measured by MTS cell proliferation colorimetric assay kit on a microplate reader (Infinite M200, Tecan, Switzerland) at 490 nm. The following formula was used: Cell viability % = (A_ treated_ - A_ background_) / (A_ control_ - A_ background_) × 100%, where the A_ treated_ was the absorbance value of the cells with treatments, A_ control_ was the absorbance value of the cells without treatments and A _background_ was the absorbance of the medium without cells. Each assay was repeated for 5 times, and the concentration-viability curves were made and *IC_50_* values were calculated by Origin 8.1 software. The biocompatibility and biosafety of the peptide ligands (Ang2 and EP-1) and the blank carriers were also evaluated in U87-MG and HBMEC cells with the same procedure.

Meanwhile, the short-term cytotoxicity of different DOX formulations at high concentration to HBMEC cells was performed by MTS assay *in vitro*. HBMEC cells were seeded into 96-cell plates at a density of 1 × 10^4^ cells/well and cultured for 24 h at 37 ℃. Then different DOX formulations were added at a DOX concentration of 20 μM. After incubating for 3 h, cell viability was determined by MTS assay.

### 2.13 Biosafety evaluation of the dual-functional dendrimer-based carrier

Biosafety of the dual-functional dendrimer-based vehicles was evaluated by hemolysis assay. Briefly, mouse whole blood was centrifuged at 1000 g for 5 min and washed five times by PBS to obtain pure erythrocytes. Then 500 μL of 4% erythrocytes (v/v) was mixed with 500 μL of the dual-functional dendrimer at various concentrations (0, 0.05, 1.25, 6.25 and 31.25 μM). The mixtures were put into an incubator shaker with the condition of 37 ℃ and 100 rpm. After being incubated for 8 h, the samples were centrifuged and the absorbance of the supernatants at 54 nm was detected and recorded using a UV-Vis spectrophotometer (). Erythrocytes mixed with deionized water were used as 100% hemolysis. The percentage of hemolysis was calculated following the equation: Hemolysis (%) = A/A_0_ × 100%, where A represents the absorbance of supernatant for erythrocytes with P4PEA, and A_0_ is the absorbance of erythrocytes after complete hemolysis in pure water.

The biosafety of the dual-functional dendrimer was further determined by AM/PI co-stained studies. HBMEC cells were seeded into 96-well plated at a density of 5000 cells per well and incubated for 24 h. Then the P4PEA was added into the cells at various concentration (0, 0.05, 0.25, 1.25, 6.25, 31.25 μM). After another 24 h incubation, the cells were washed with PBS for three times and co-stained by a Calcein-AM/PI Double Stain Kit (KeyGEN BioTECH Co., Ltd. Jiangsu, China). Finally, the cells were observed and recorded on LSCM at the channel of 488 nm and 543 nm.

### 2.14 Cellular uptake* in vitro*

Intracellular uptake of DOX was detected using flow cytometry firstly. In the brief, HBMEC and U87-MG cells were seeded into 6-cell culture plates at a density of 5 × 10^5^ cells/well and cultured for 24 h. Then different DOX formulations were added into the cells at a DOX concentration of 20 μM, respectively. Cells without any drugs were used as spontaneous fluorescence control. After incubating for 1 h, the medium was removed and the cells were washed with cold PBS for three times and harvested. Then the cells were analyzed by BD Accuri^TM^ C6 system (BD, USA).

The intracellular localization of DOX in HBMEC and U87-MG cells was further detected by confocal imaging. Cells were cultured in the glass bottom dishes at a density of 2 × 10^5^ cells/dish for 24 h, and then the cells were treated with DOX-loaded different carriers at a DOX concentration of 10 μM for 2 h. Afterwards, the cells were washed with cold PBS for three times and fixed with 4% (v/v) paraformaldehyde. Then the cells were stained with Hoechst for 15 min before observing by laser scanning confocal microscope (Zeiss 710, Germany).

### 2.15 Subcellular localization

HBMEC and U87-MG cells were seed into glass bottom dishes with the density of 5 × 10^4^ cells per dish and cultured at 37 ℃ for 24 h. Then Cy5.5-labeled different carriers were added into the cells at a final concentration of 1 μM. After incubating for 2 h, the medium was removed and washed with PBS for three times. Then the lysosomes were stained by LysoTracker Green (0.1 μM) for 1 h at 37 ℃. Hoechst was used to stain the nucleus. Then the subcellular distribution was recorded on LSCM at the channel of 405 nm, 488 nm and 633 nm.

### 2.16 Establishment of the BBB model *in vitro*

HBMEC monolayer model was established to study the BBB transportation of the dual-targeting drug delivery system according to the protocol reported previously 47. Briefly, 2% gelatin was pre-coated on transwell inserts (12-well Polycarbonate Membrane Transwell Insert of 1 μm mean pore size, corning, NY, USA) for 30 min at 37 ℃. Then HBMEC were seeded at a density of 2 × 10^4^ cells per well and cultured for 4 days. The medium was changed every two days and the Trans Endothelial Electrical Resistance (TEER) was measured every day. The BBB model was examined by 4 h of permeation assay and TEER values. Only both the TEER value of the BBB model was over 250 Ω/cm^2^ and the medium did not leak in 4 h, the establishment of BBB model was successful and could be used for the further experiments.

### 2.17 Transport assay across the BBB *in vitro*

To evaluate the ability of DOX across the BBB after encapsulating into the carriers, free DOX, P4PD, P4PAD, P4PED and P4PEAD were added into the corresponding inserts of the BBB model *in vitro* with the DOX concentration of 20 μM and cultured at 37 ℃. Then a volume of 400 μL medium was taken out from the acceptor compartments at 30, 60, 90, 120, 180 min and 400 μL fresh medium was supplied immediately. The amounts of DOX transported across BBB were determined using fluorescence spectrophotometer with the excitation wavelength of 480 nm and emission wavelength of 590 nm.

### 2.18 Dual-targeting effects *in vitro*

To evaluate the dual-targeting effect of the dual-functionalized carrier *in vitro*, a HBMEC and U87-MG cells co-culture model was established. U87-MG cells were seed into 12-well plates at a density of 2 × 10^4^ cells per well and allowed to culture at 37 ℃ for 48 h. The BBB model *in vitro* was established as described above. Then the inserts with monolayer HBMEC cells were transferred to the culture plates with confluent U87-MG cells. The medium of the donor inserts was removed and 500 μL of free DOX, P4PD, P4PAD, P4PED and P4PEAD solution was added at the DOX concentration of 20 μM, respectively. After 3 h incubation, the inserts were removed and U87-MG cells in 12-well plates were cultured for another 24 h. Then the cell viability of U87-MG cells was determined by the MTS assay. Additionally, the same model and procedure were performed as described above. After incubating for 3 h, the inserts were removed and U87-MG cells in 12-well plates were collected by trypsin. Then the DOX uptake by U87-MG was detected by flow cytometry.

### 2.19 Dual-targeting effect *in vivo*

To investigate the BBB penetrating and the glioma targeting effect of the dual-targeting dendrimer *in vivo*, the glioma-bearing mouse model was established in female CB-17 SCID mice. The female CB-17 SCID mice were obtained from Charles river and raised separately at 22 ± 2 ℃ under rotating 12 h light/dark condition. The animals were given immunodeficiency mouse feed and sterile water. Orthotopic glioma model was established according the previous report 54. Briefly, the female CB-17 SCID mice with the average body weight of 18 g were deeply anesthetized by 1 wt% pentobarbital sodium at the dose of 100 mg/kg. Then the cranium was exposed by midline sagittal incision. Subsequently, a burr hole of 1 mm in diameter was drilled at the right striatum (0.5 mm anterior and 0.5 mm lateral from the bregma) using a stereotactic fixation device (Stoelting, USA). Approximate 2 × 10^5^ U87-MG cells in 5 μL PBS were stereotaxically implanted into the striatum at a depth of 3 mm from the brain surface. The scalp incision was closed with bone wax covering. Meanwhile, the Cy5.5 labeled dual-functionalized carrier was prepared by reacting the G4 PAMAM with Cy5.5-NHS before the establishment of the carrier. Cy5.5 labeled other formulated carriers was also prepared as comparison. At ten days after inoculation, the Cy5.5 labeled nanocarriers with different modification were injected into the mice via the tail vein with the Cy5.5 dose of 2 mg/kg. The fluorescence signals of Cy5.5 labeled different carriers which crossing the BBB and targeting into glioma site were recorded at 6 h, 12 h, 24 h and 48 h using the *in vivo* imaging system. Then the mice were sacrificed at the end point and the main organs (brain, heart, liver, spleen, lung and kidney) were excised. The fluorescent signal in different tissues was recorded on the *in vivo* imaging system.

### 2.20 Therapeutic efficacy* in vivo*

Therapeutic efficacy of DOX after incorporating into the carriers was evaluated in glioma bearing female BALB/c nude mice. The mice were obtained from Charles river and raised in the same condition mentioned above. The Orthotopic glioma model was established through the same protocol described above. At day 3 after tumor inoculation, the mice were divided into six groups (9 mice per groups) randomly. Animals in blank control group were administrated with saline. Other five groups were treated with free DOX, P4PD, P4PAD, P4PED and P4PEAD *via* tail vein with a DOX dose of 5 mg/kg, respectively. Administrations were made every two days with total four doses per mouse. At day 15, three mice of each group were sacrificed and the main organs (liver, kidney, heart, lung, spleen and brain) were dissected and fixed in 4% paraformaldehyde solution for 48 h before being embedded in paraffin. Then the sliced organ tissues mounted on the glass slides were stained by hematoxylin and eosin (H&E) and observed by a digital microscope. The remained six mice were maintained and recorded the survival carefully until all the mice dead. The Kaplan-Meier survival curves were plotted by GraphPad Prism (GraphPad software Inc.) for each group.

### 2.21 Statistical analysis

Data are presented as mean ± standard deviation, except that of survival times. All the experiments were carried out in or over triplicate and Student's t-test was performed to assess statistical significance of the results (*P < 0.05, **P < 0.01, ***P < 0.001 and ****P < 0.0001).

## 3. Results and Discussion

### 3.1 Screening EGFR-targeting peptide from OBOC combinatorial library

As EGFR has been widely found to be overexpressed in glioma and has been used as a diagnostic and prognostic marker for glioma 3, we chose it as a marker for targeting drug delivery. High-affinity peptides targeting EGFR were identified by high throughput screening from a peptide combinatorial library using “one-bead one-compound” (OBOC) method with the assistance of microfluidic technology. OBOC approach is one of the most popular methods for peptide screening from the combinatorial library. However, the traditional procedure of OBOC method is time-consuming and has high false positive rate, limiting its screening efficiency 55. This has been overcome by an integrated microfluidic screening system in which the positive beads are separated in a magnetic field and the candidate molecules are sequenced *via* on-chip mass spectrometry 50, 51. A 10-mer OBOC peptide library towards EGFR was designed and constructed through solid phase peptide synthesis (SPPS) strategy (**Scheme S1**). The peptide library was designed with the sequence of X_1_X_2_X_3_X_4_X_5_X_6_X_7_X_8_X_9_G in which each X represented any of the four different amino acid residues to improve the diversity of the library. Hydrophobic and alkaline amino acid residues designed at N-terminal might improve the hydrophobic and electrostatic interactions between peptide ligands and HER family 56. The glutamic acid was elongated at C-terminal to reduce the steric hindrance during the construction of the peptide library. A capacity of approximately 2.6 × 10^5^ was achieved in this library **(Scheme S1)**. Each bead in the library would be randomly distributed with one peptide through the “split and poor” approach. Biotinylated EGFR was incubated with the beads to recognize the beads with peptides that had high affinity to EGFR. The positive beads were then labeled with SA-coated magnetic beads and were separated in a magnetic microfluidic chip. The positive peptides on the trapped beads were subsequently collected and identified by *in situ* matrix assisted laser desorption/ionization time-of-flight mass spectrometry (MALDI-TOF-MS) using “one-well one-bead” strategy **(Scheme S2)**. Consequently, a total of 168 positive peptide sequences were identified. Sequence alignment showed that the most frequent amino acid residues in each position hit the high probability interaction between peptide and EGFR protein. We found that alkaline and acidic amino acids in the first two positions of N-terminal might enhance the affinity of peptide ligands to EGFR according to the multiple sequence alignment analysis (**Figure S1**). Therefore, we redesigned five peptides, reference here as EGFR-targeting peptide (EP-1 to EP-5), according to the most frequent matches among all residues to further screening (**Table S1**). The reasonable elongation of cysteine residue at the C-terminal was for the covalent immobilization of the peptides to the gold-coated chip for subsequent peptide screening through surface plasmon resonance imaging (SPRi).

**Figure 1:**
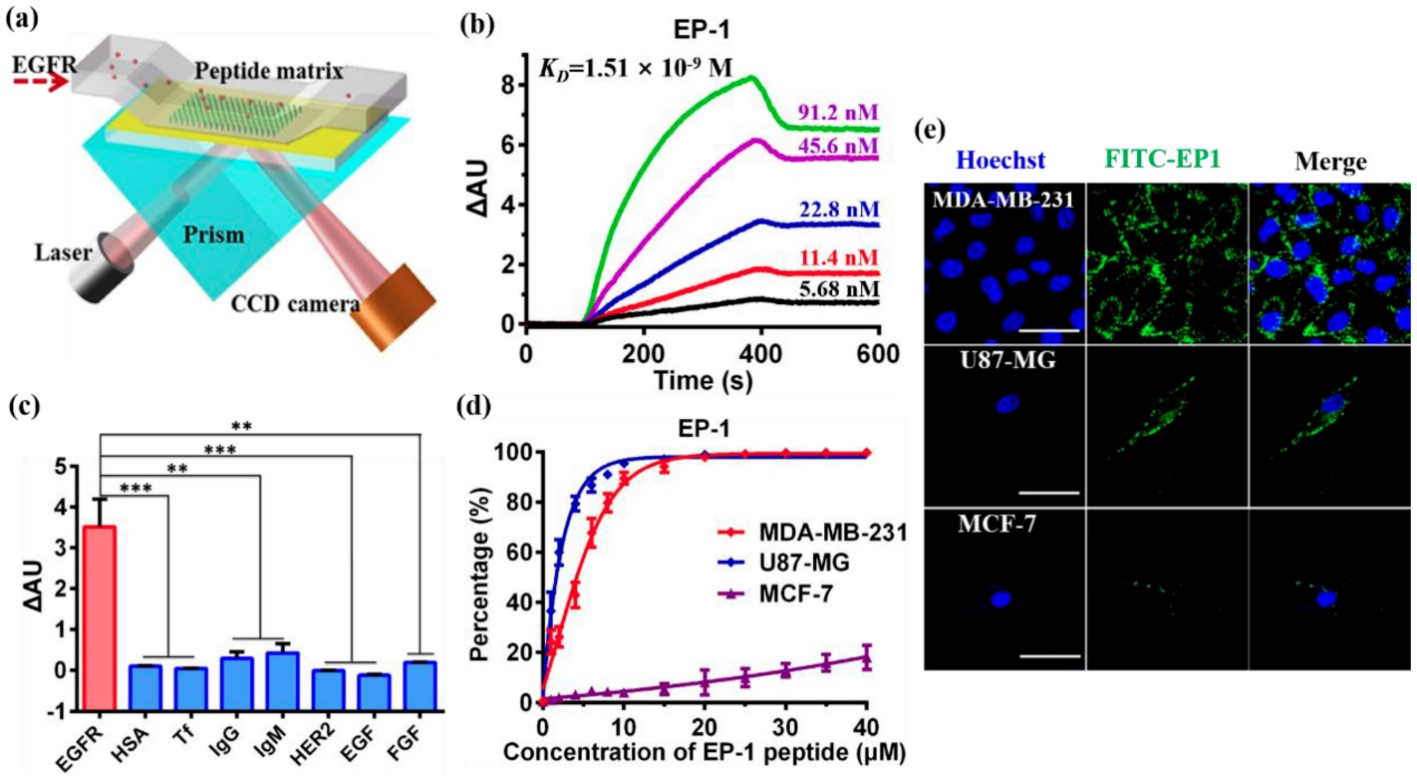
Evaluation of the affinity and specificity of peptide EP-1 towards EGFR. (a) Schematic illustration of high-throughput peptide library screening using surface plasmon resonance imaging (SPRi). The synthesized thiol-containing peptides were immobilized on the gold-coated SPRi chip. EGFR was passed through the flow chamber at various concentrations to check its binding with the peptides. (b) Representative SPRi sensorgram shows the binding of EP-1 to different concentrations of EGFR. The *K_D_* value was determined to be 1.51 × 10^-9^ M using BIAevaluation version 4.1 software (Biacore, Inc.). (c) SPRi binding signals of EP-1 towards EGFR, HSA, Tf, IgG, IgM, HER2, EGF and FGF. Error bars represent the standard deviation (n = 3). **p < 0.01, ***p < 0.001 (Student's t-test). (d) Binding affinity of EP-1 towards EGFR in MDA-MB-231, U87-MG and MCF-7 cell lines. The percentage of cells bound with FITC-labeled EP-1 was detected by flow cytometry. Error bars represent the standard deviation (n = 3). (e) Confocal microscopic images showing the binding of FITC-labeled EP-1 to MDA-MB-231 (upper), U87-MG (middle) and MCF-7 (bottom). Cells were incubated with 40 μM of FITC-EP1 for 1 h. Scale bar: 50 μm.

The binding affinity between the redesigned peptides and EGFR protein was determined by SPRi, a real-time, label-free and high-throughput sensor technique that could detect the molecular binding occurring close to the SPR-active metal surface by monitoring the refractive index changes 57. The peptides were immobilized on the chip through covalent binding between the thiol group and the gold surface, the binding affinity between the peptides and EGFR was evaluated by checking their interaction over a range of EGFR concentration (**Figure 1a**). Kinetic analysis showed that the equilibrium dissociation constant (*K_D_*) values of EP-1, EP-3, EP-4, and EP-5 were of the same order of magnitude while the *K_D_* value of EP-2 was significantly lower **(Figure 1b, Figure S2, Table S2)**, indicating the low binding ability between EP-2 and EGFR. We therefore chose EP-1, EP-3, EP-4, and EP-5 as the high-affinity peptide candidates.

We further investigated the specificity of these peptide candidates to EGFR. The binding affinity between the peptides and a series of proteins including human serum albumin (HSA), transferring (Tf), immunoglobulin G (IgG), immunoglobulin M (IgM), human epidermal growth factor receptor-2 (HER2), epidermal growth factor (EGF), fibroblast growth factor (FGF) and EGFR were compared. SPRi analysis showed that the binding signal of EP-1 to EGFR was significantly higher (~3.52 ΔAU) than to the other proteins (< 0.43 ΔAU for IgM and IgG while negligible for HSA, Tf, HER2, EGF and FGF, **Figure 1c**), suggesting the high specificity of EP-1 to EGFR. In the contrast, EP-3 and EP-4 exhibited relatively low binding affinity and poor specificity with EGFR (**Figure S3a, b**). EP-5 peptide exhibited significantly higher binding signal with EGFR than with HSA, Tf, IgG, IgM HER2, EGF, and FGF. However, it has high binding signal with IgM, an abundant protein in the blood (**Figure S3c**). Therefore, we chose EP-1 as the molecular probe for targeting EGFR in the glioma.

We further investigated the binding affinity and specificity of peptide EP-1 to EGFR in cell lines with different EGFR expression, including glioblastoma cell line U87-MG, triple-negative breast cancer cell line MDA-MB-231 and human breast adenocarcinoma cell line MCF-7. The high expression of EGFR in U87-MG and MDA-MB-231 cells and low expression of EGFR in MCF-7 cells have been reported in the previous report 3, 58. Thus, we evaluated the expression of EGFR in the cell lines by flow cytometry analysis firstly. As expected, MDA-MB-231 (99.6%) and U87-MG (99.9%) exhibited high expression level of EGFR, MCF-7 exhibited low expression of EGFR (6.5%) (**Figure S4, up**), in accordance with the previous reports 3, 58. These results were confirmed by confocal images revealing the immunostaining of the cells with fluorescent-labeled anti-EGFR which also showed high expression of EGFR in U87-MG and MDA-MB-321 while low expression of EGFR in MCF-7 (**Figure S4, bottom**). We then investigated the binding of EP-1 to EGFR over a range of EP-1 concentrations using U87-MG and MDA-MB-231 as the positive control and MCF-7 as the negative control. Kinetic analysis of the binding of EP-1 to the cells was performed using flow cytometry analysis. As expected, the binding of EP-1 to U87-MG and MDA-MB-231 increased rapidly with increasing concentration of EP-1 and reached a plateau of ~100% at 20 μM of EP-1(**Figure 1d**), showing the saturation binding of EP-1 to these two cell lines with high expression of EGFR that is characteristic of ligand-receptor binding. On the contrast, the binding of EP-1 to MCF-7 that had low expression of EGFR increased slowly as the increasing concentration of EP-1 and only reached ~18.0% even at 40 μM for EP-1 without reaching saturation (**Figure 1d**). These results were also confirmed by confocal images showing the binding of fluorescent labeled EP-1 in U87-MG and MDA-MB-231, but not in MCF-7 (**Figure 1e**), which again indicated the high affinity and specificity of EP-1 towards EGFR in cell lines. These results, together the high affinity and specificity of EP-1 assessed at the molecular level, demonstrated the potential of EP-1 as a promising probe for targeting EGFR in the drug delivery system.

### 3.2 Fabrication and characterization of the dual-targeting carrier

The dual-targeting drug delivery system was established by conjugating the fourth generation PAMAM dendrimer (P4) with EP-1 that targeted EGFR in the glioma and Ang2 that targeted LRP1 in the BBB (**Scheme 1a**). PAMAM dendrimer was firstly modified with PEG by reacting the primary amino groups of the dendrimer with the NHS groups of Mal-PEG-NHS (MW = 2000) and mPEG-NHS (MW = 750). Mal-PEG-NHS was used as crosslinkers for the further conjugation of peptide ligands and the mPEG-NHS was used as functional conjugate to shield the positive charge of the dendrimer. Afterwards, the peptide ligands were conjugated on PAMAM through Michael addition reaction between the thiols of the peptides and the Mal groups of the heterobifunctional PEG chain.

**Figure 2:**
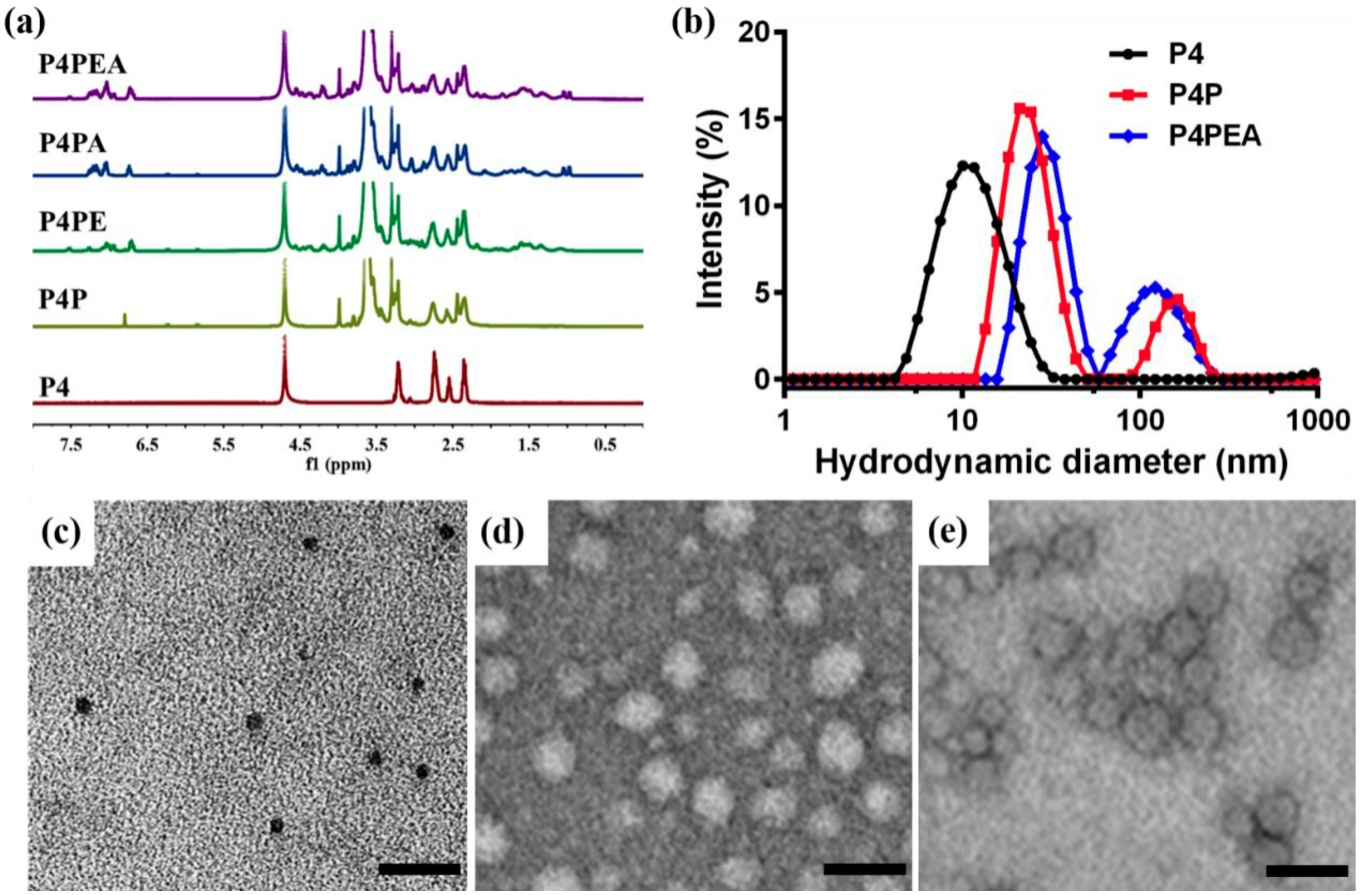
Characterization of the dendrimer-based carriers. (a) ^1^H NMR spectrum of G4 PAMAM (P4, brown), PEGylated PAMAM (P4P, olive), PAMAM-PEG-EP1 (P4PE, green), PAMAM-PEG-Ang2 (P4PA, blue) and PAMAM-PEG-EP1-ANG2 (P4PEA, purple) in D_2_O. (b) Size distribution of the dendrimer-based carriers characterized by DLS. (c-e) Morphological characterization of (c) P4, (d) P4P and (E) P4PEA by TEM.

The chemical structure of the dendrimer-based carriers was characterized by ^1^H NMR spectroscope using D_2_O as solvent (**Figure 2a**). The multiple peaks between 2.2 and 3.3 ppm were the corresponding peaks of PAMAM dendrimer, the solvent peak of D_2_0 was recorded at 4.7 ppm (**Figure 2a, brown peak**). The new triplet peaks at 5.82-5.86 ppm and 6.21-6.25 ppm corresponded to the proton of imido groups conjugated with carbonyl of PEG chains (-*NH*-C=O-), which indicated the successful modification of PAMAM dendrimer by PEG (**Figure 2a, olive peak**). As a result of the integral analysis of the proton signal of methylene next to the amide group (-*CH_2_*-CO-NH-, 2.20-2.45 ppm) in PAMAM dendrimer and the integral of the proton signal of methoxyl (a sharp peak at 3.3 ppm) in the short chain PEG, the grafting ratio of short chain PEG on the PAMAM periphery was determined to be 22 on average. Meanwhile, according to the integral of the proton signal at 3.71 ppm (-*CH_2_CH_2_*O-) of the main PEG chains, 3.3 ppm (methoxyl peak) of the short PEG chains and the number of the PEG arms in the PEG chains, about 15 bifunctional PEG chains were calculated to be successfully conjugated on the surface of PAMAM dendrimer. Additionally, a small peak appeared at 6.79 ppm related to the Mal group of the bifunctional PEG, which suggested the potential of the PEGylated dendrimer for further conjugation of other ligands or functional molecules. Compared with the ^1^H NMR spectrum of the PEGylated dendrimer (P4P), a new sharp peak at 7.52 ppm and several new multiple peaks at 6.56-7.30 ppm appeared in PAMAM-PEG-EP1 (P4PE) and PAMAM-PEG-EP1-Ang2 (P4PEA) (**Figure 2a, green and purple peak**), which were in accordance with the characteristic peaks of EP-1 peptide (**Figure S5b**). Meanwhile, a new shoulder peaks at 0.96-1.04 ppm which represented the characteristic peaks of Ang2 peptide (**Figure S5a**) were also found in PAMAM-PEG-Ang2 (P4PA) and P4PEA (**Figure 2a**, **blue and purple peak**). These results indicated the successful modification of the dendrimer by the peptide ligands.

Size distribution, morphology and electriferous properties of the dendrimer-based carriers were characterized by dynamic light scattering (DLS), transmission electron microscope (TEM), and zeta-potential measurements. DLS analysis showed that the hydrodynamic diameter of the dendrimer-based carriers increased from ~5.78 nm for G4 PAMAM (P4) to ~11.63 nm for PEGylated PAMAM (P4P0 and ~16.80 nm for P4PEA (**Figure 2b**). Although the particles in ~158.6 nm (P4P) and ~127.6 nm (P4PEA) gave relatively high scattering intensity in solution, most of carriers still stayed at the small diameter because the scattering intensity was proportional to the six power of the particle size. TEM images showed that the dendrimer-based carriers were spherical mono-dispersion, rather than large aggregates (**Figure 2c-e**). We measured the size of the particles in TEM images an found that the size distributions were determined to be 7.03 ± 1.04 nm for P4, 20.2 ± 3.63 nm for P4P and 26.07 ± 8.15 nm for P4PEA (**Figure S6**), in the similar range of the ones assessed by DLS. Meanwhile, the particle size changed negligible within 72 h both in water and PBS, which demonstrated the well stability and dispersibility of the dendrimer-based carriers in aqueous solution (**Figure S7**). Considering that a drug carrier with the size < 100nm would cross the BBB efficiently 47, while a carrier < 5 nm would be rapidly eliminated by the kidney 59, we suspected that our dual-targeting dendrimers would exhibit efficient BBB-penetrating, long-circulating and enhanced tumor-accumulating properties.

Zeta potential measurement showed that the surface potential of P4, P4P and P4PEA was 21.0 ± 1.0 mV, 4.1 ± 0.3 mV, 6.5 ± 0.1 mV, respectively. The modification of PEG chain reduced the positive charge greatly due to the shielding effect of the PEG, which would reduce the cytotoxicity and biotoxicity of the carriers. Meanwhile, the residual positive charge would benefit BBB penetrating of the carriers by adsorption-mediated transcytosis 45.

### 3.3 Drug loading and *in vitro* release

**Figure 3:**
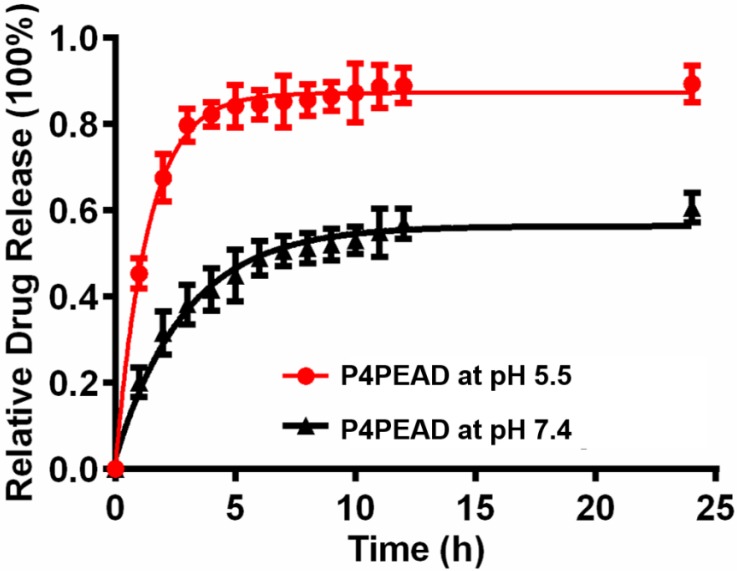
*In vitro* drug release of P4PEAD at pH 5.5 and pH 7.4. Error bars represent the standard deviation (n = 3).

The dendrimer-based dual-targeting carrier had abundant interior cavities which could encapsulate therapeutic regents by physical interaction such as hydrophobic interaction and electrostatic interaction 60, 61. Doxorubicin hydrochloride was changed into the hydrophobic form by triethylamine and was encapsulated into the dendrimer due to the relative hydrophobicity of the dendrimer cavities. The drug loading efficiency (LE%) and encapsulation efficiency (EE%) of the carriers were determined to be 4.3% and 58.9%, respectively by UV-visible scanning spectrophotometer. *In vitro* release behaviors of the encapsulated DOX were investigated at pH 7.4 and pH 5.5 that mimicked the physiological and pathological pH environment. Under the physiological environment (pH 7.4), DOX released slowly and reached a plateau of 50% after 10 h, while at pH 5.5 that mimicked the weak acidic microenvironment of tumor, the release rate increased rapidly and reached 80% within 4 h (**Figure 3**). These results suggested that the release of DOX could be controlled using pH as a trigger. Under the physiological condition, the hydrophobic interaction between DOX and the interior of dendrimer was strong enough to retain the 'dense core' conformation (with the maximum density at the dendrimer core and uniform void spacing), which prevented the drug from leaking out. When the environment changed to low pH, the conformation of the dendrimer-based drug carrier changed from a 'dense core' to a 'dense shell' (with a maximum density at the periphery but non-uniform void spacing) because of the ion pairing, which reduced the interaction between the drugs and dendrimer and accelerated the drug release 62. The controllable drug release of this dual-targeting carrier in the pathological environment would effectively increase the accumulation of drugs in the tumor site and enhance the anti-tumor efficacy. The limited drug leakage during circulation would reduce the toxicity and side effect of drugs for the normal tissues.

### 3.4 *In vitro* evaluation of cytotoxicity and intracellular uptake

**Figure 4:**
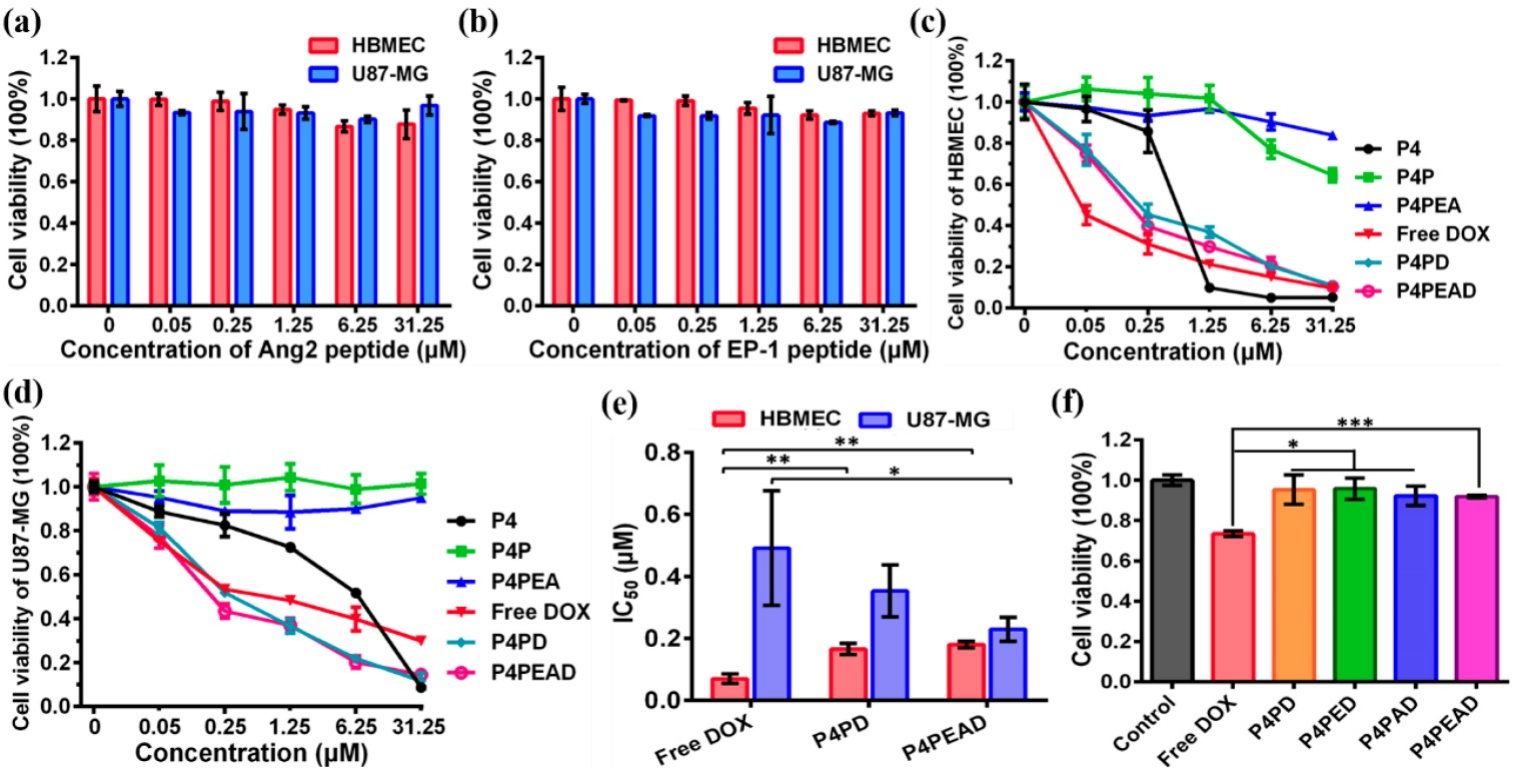
*In vitro* evaluation of biocompatibility and antitumor efficacy of the dual-targeting drug delivery system. (a, b) Cytotoxicity of peptides (a) Ang2 and (b) EP-1 to HBMEC and U87-MG cells. (c, d) Cytotoxicity of blank dendrimer-based carriers (P4, P4P and P4PEA), free DOX, P4PD and P4PEAD to (c) HBMEC and (d) U87-MG cells. The cells viability was checked by MTS assay after incubating the cells with different concentrations of peptides, blank carriers and DOX-loaded dendrimer carriers for 48 h. Error bars represent standard deviation (n = 5). (e)* IC_50_* values of different DOX-loaded dendrimers fitted by Origin 8.1 software. Error bars represent standard deviation (n = 5). (f) Short-term cytotoxicity of different DOX formulations to HBMEC cells after incubating the cells with the DOX formulations for 3 h at the DOX concentration of 20 μΜ. Error bars represent standard deviation (n=5). *p < 0.1, **p < 0.01, ***p < 0.001 (Student's t-test).

To evaluate the biocompatibility and biosafety of the dual-targeting drug delivery system, the cytotoxicity of the peptide ligands (EP-1 and Ang2) and the blank dendrimer-based carriers (P4, P4P and P4PEA) without encapsulating DOX in HBMEC and U87-MG cells was investigated by [3-(4, 5-dimethylthiazol-2-yl)-5-(3-carboxymethoxyphenyl)-2-(4-sulfophenyl)-2H-tetrazolium (MTS) cell proliferation colorimetric assay. We found that after incubating the HBMEC and U87-MG with Ang2 or EP-1 over a range of peptide concentrations for 48 hours, the viability of these two cell lines remained ~90% (**Figure 4a and b**), which revealed the biosafety of the peptides as targeting probes. G4 PAMAM dendrimer (P4) without any modification exhibited high toxicity to both of the cells due to its cationic surface (**P4, Figure 4c and d**). The toxicity of P4 in HBMEC cells was much higher than that in U87-MG cells due to higher tolerance of tumor cells than normal cells. PEG modification significantly decreased the cytotoxicity of the dendrimer for HBMEC and U87-MG cells benefiting from the charge shielding effect of PEG (**P4P, Figure 4c and d**). Over 80% of U87-MG cells and 60% of HBMEC cells were survival after exposing the cells to P4P for 48 h at the concentration of 31.25 μM. Functionalization of the carriers with the peptide ligands further reduced the cytotoxicity of the dendrimer so that more than 85% of the cells were alive over a range of peptide concentrations (**P4PEA, Figure 4c and d**). Besides, the biocompatibility and biosafety of the dual-targeting dendrimer was also examined by hemolysis assay and Calcein-AM/PI co-stained assay. As shown in **Figure S8a**, no significant hemolysis (less than 8%) was found in the presence of P4PEA for 8 h. Besides, strong green fluorescence signal and negligible red fluorescence signal were recorded after being treated with P4PEA detected by Calcein-AM/PI co-stained assay, which demonstrated the low cytotoxicity of the dual-targeting dendrimer (**Figure S8b**). These results demonstrated the good biocompatibility and biosafety of the peptide-functionalized dual-targeting dendrimer vehicles.

We then evaluated the anti-proliferation effect of different DOX formulations against HBMEC and U87-MG cells by MTS assay (**free DOX, P4PD and P4PEAD, Figure 4c and d**). The viability of the HBMEC increased at low DOX concentration when DOX was encapsulated in the dendrimer compared to the free DOX (**Figure 4c**), indicating reduced cytotoxicity of DOX to the normal cells by the dendrimer-based carrier. However, at high DOX concentration, the viability of HBMEC treated with the dendrimer-encapsulated DOX reduced to the same level as the one treated with free DOX. This might be due to the released DOX during the experimental period reached to the effective inhibition concentration for HBMEC cells. The viability of the tumor cells U87-MG significantly decreased when incubating with DOX-encapsulating dendrimer (P4PD and P4PDEA) compared to free DOX (**Figure 4d**), indicating the increased anti-proliferation effect of DOX induced by the dendrimer-based drug carrier. We further calculated the half maximal inhibitory concentration (*IC_50_*) of the cells. DOX displayed an inhibited cytotoxicity to the normal cells HBMEC after being loaded into dendrimer compared to free DOX (**Figure 4e**, *IC_50_* was determined to be 0.07, 0.17 and 0.18 μM for free DOX, P4PD and P4PEAD, respectively), suggesting that limited DOX released from the dendrimer-based carriers and diffused into the nuclei of the normal cells. On the contrary, DOX exhibited higher cytotoxicity for U87-MG cells in the presence of the carriers compared with free DOX (**Figure 4e**, *IC_50_* was determined to be 0.49, 0.36 and 0.23 μM for free DOX, P4PD and P4PEAD, respectively), suggesting that the carriers could improve the intracellular uptake of DOX and the controllable release of the drugs in a short time. The carriers modified with the peptide ligands further increased the cytotoxicity of DOX significantly to U87-MG compared with the non-modified dendrimer, indicating the enhanced cellular uptake of therapeutics due to the peptide-induced targeting effect.

The short-term cytotoxicity of different DOX formulations against HBMEC cells was evaluated to eliminate the interference of the cytotoxicity of the drug delivery system to HBMEC cells during transporting BBB *in vitro* (**Figure 4f**). As expected, free DOX exhibited high cytotoxicity to HBMEC cells (cell viability of 73.4%) after 3 h's incubation. However, when DOX was loaded into the dendrimers, the cell viability of HBMEC excessed 90% (**Figure 4f**), indicating that the dendrimer significantly reduced the cytotoxicity of DOX. These results also suggested that the *in vitro* BBB model established on the basis of HBMEC cells could keep integral when incubating with the dendrimers, confirming the reliability of the *in vitro* BBB model.

We further investigated the targeting effect of the dual-targeting carriers through quantifying the intracellular uptake of the drugs by HBMEC and U87-MG cells. We first evaluated the expression of EGFR and LRP1 in U87-MG and HBMEC cells. Flow cytometry analysis showed that the expression of EGFR was high (99.6%) in U87-MG while low (21.5%) in HBMEC cells, in accordance with the confocal imaging showing the overexpression of EGFR in U87-MG cells while negligible expression of EGFR in HBMEC cells (**Figure S4b and S9a**). Meanwhile, the expression of LRP1 was high (89.6%) in HBMEC and moderate (53.7%) in U87-MG cells (**Figure S9b, c**), in accordance with the previous reports showing high expression of LRP1 in HBMEC cells 38.

**Figure 5:**
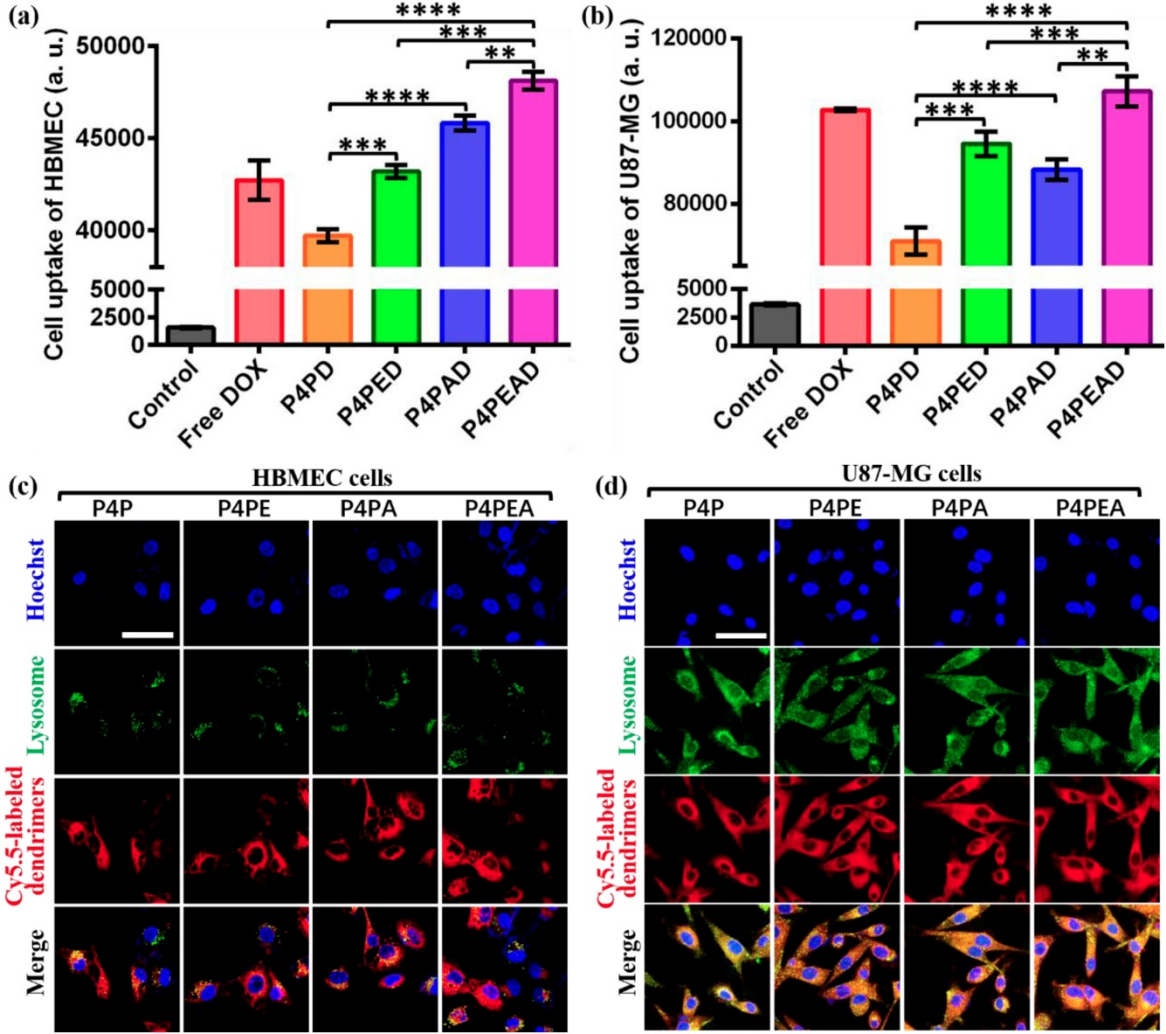
(a, b) Intracellular uptake of different DOX-loaded dendrimers by (a) HBMEC and (b) U87-MG cells detected by flow cytometry. Dendrimers were incubated with the cells for 2 h before flow cytometry measurement. Cells without treatment were used as control. Error bars represent standard deviation (n=5). **p < 0.01, ***p < 0.001, ****p < 0.0001 (Student's t-test). (c, d) Subcellular trafficking of different DOX formulations in (c) HBMEC and (d) U87-MG cells detected by LSCM. Cells were incubated with Cy5.5-labeled different dendrimers for 2 h at the dendrimer concentration of 1 μM. LysoTracker Green DND-26 was used to stain lysosomes at the concentration of 0.1 μM. Hoechst was used to stain nucleus. Scale bar: 50 μm.

We then investigated the intracellular uptake of DOX from different DOX formulations. After incubating the cells with different DOX formulations for 2 h at the DOX concentration of 20 μM, the fluorescent intensities of the cells were investigated by flow cytometry. As expected, the fluorescent intensity of the cells was significantly higher in U87-MG than in HBMEC (**Figure 5a**), demonstrating the targeting effect of the peptide-functionalized dendrimers to the tumor cells. The fluorescent intensity of HBMEC was significantly higher after incubating the cells with the Ang2-functionalized dendrimers compared to the nonfunctionalized and EP-1 modified ones (**Figure 5a**), indicating the effective Ang2-induced targeting effect of the dendrimers to the cells with high expression of LRP1. EP-1-modified dendrimers exhibited higher fluorescent intensity in HBMEC than the non-modified dendrimers, and the dual-functionalized dendrimers exhibited the highest fluorescent intensity (**Figure 5a**). This was due to the moderate expression of EGFR (21.5%) in HBMEC. The cellular uptake of DOX in U87-MG was in the order of P4PEAD > DOX > P4PED > P4PAD > P4PD (**Figure 5b**), consistent with the overexpression of EGFR and the moderate expression of LRP1 in U87-MG cells. These results demonstrated the targeting effect induced by Ang2 and EP-1 peptides. They also showed that the dual-functionalized dendrimer significantly enhanced the intracellular uptake by the ligand-receptor mediated endocytosis.

The intracellular disposition of DOX in HBMEC and U87-MG cells was further determined by confocal imaging after incubating the cells with different DOX formulations for 2 h. As expected, almost all the free DOX penetrated into the cells and accumulated in the nuclei in both cells (**Figure S10**). However, after DOX was loaded into the dendrimer-based carriers, most DOX was found to be located in the cytoplasma in HBMEC (**Figure S10a**), indicating that the interaction between the drugs and the dendrimers was strong enough to limit the drug release in the normal cells, which largely reduced the cytotoxicity of the drugs. By contrast, in U87-MG, the dendrimer-encapsulated DOX was found to be located in the nuclei for all the formulations (**Figure S10b**), indicating the fast release of DOX from the dendrimers in the tumor cells. The strongest fluorescence intensity in these two cells after incubating with P4PEAD compared with the other DOX-loaded carriers also revealed the enhanced drug internalization mediated by the targeting effect of EP-1 and Ang2 peptide.

We further investigated the subcellular localization of the DOX-loaded dendrimers. As endosomal/lysosome uptake and escape is an important process in the nanoparticle-based drug delivery 63, we investigated the colocalization of the dendrimers and lysosome in the cells. In HBMEC cells, we found that the lysosome distributed around the nuclei with small amounts and the carriers mainly distributed in cytoplasma within 2 h after being uptake by cells (**Figure 5c**), which would slow down the drug release induced by the weak acidic condition of lysosomes. This phenomenon could decrease the cytotoxicity of drugs for normal cells and provide enough time for transcytosis of the dendrimer-based carriers in BBB model. However, the lysosomes were abundant in glioma U87-MG cells and distributed randomly in cytoplasma. Confocal images exhibited completely colocalization of the dendrimer-based carriers with the lysosomes in the U87-MG cells (**Figure 5d**). The weak acidic environment in lysosomes would accelerate the drug release from the dendrimers, then the released DOX could diffuse out of lysosomes and further diffused into the nuclei to kill the tumor cells. This process significantly improved the effective concentration of drugs and enhanced the anti-tumor effect of drugs.

### 3.5 *In vitro* Evaluation of the BBB penetration of the dual-targeting carrier

**Figure 6:**
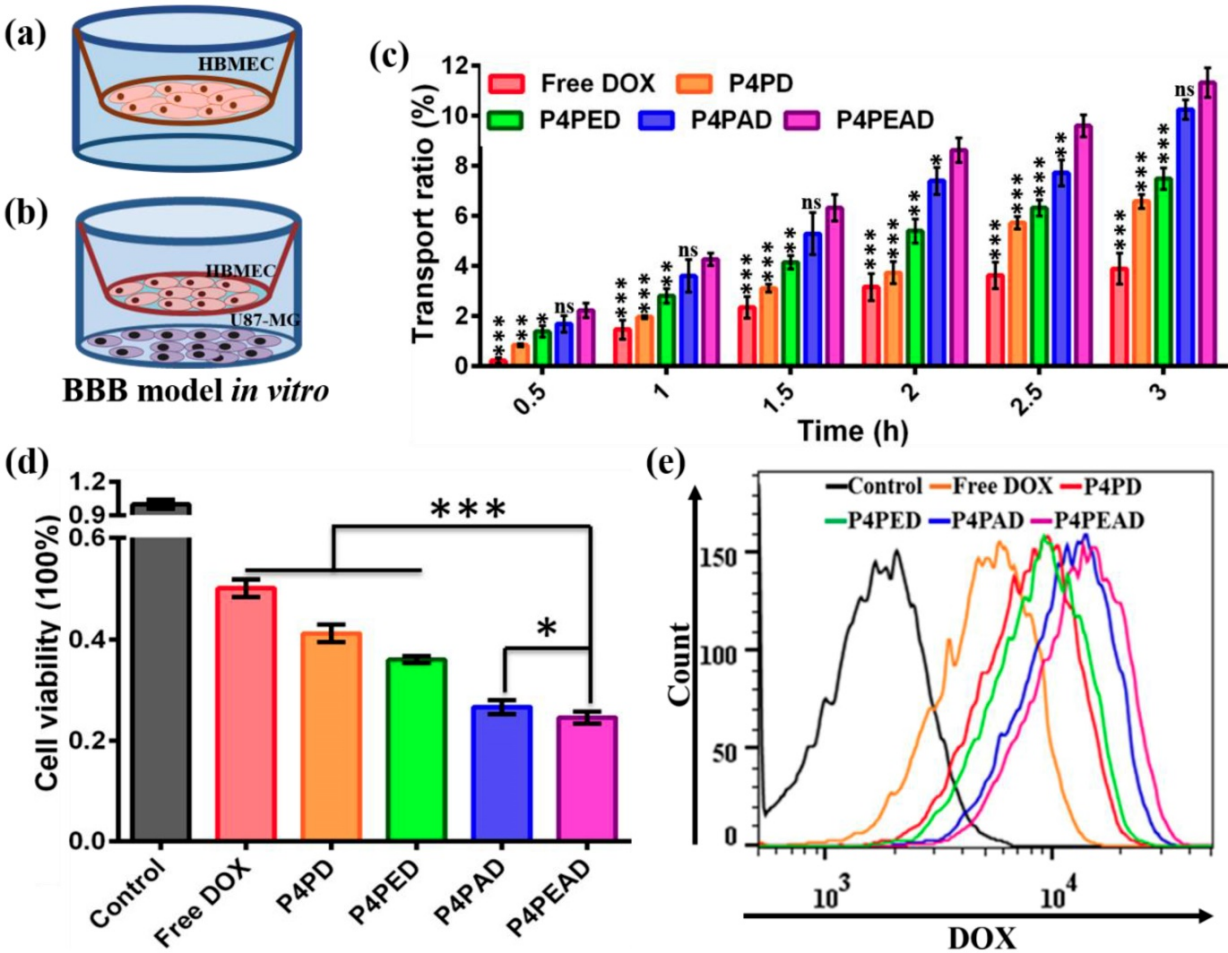
Evaluation of the BBB penetration and the dual-targeting efficacy of the DOX-loaded dual-targeting dendrimers. (a, b) Schematic illustration of the *in vitro* BBB model. (a) A monolayer of HBMEC cells were cultured on the transwell inserts, and (b) U87-MG cells were co-cultured. (c) The transport ratio of DOX across the BBB within 3 h. Error bars represent standard deviation (n = 3). *p < 0.05, **p < 0.01, ***p < 0.001 (Student's t-test). (d) The cell viability of U87-MG cells in the co-cultured BBB model. Error bars represent standard deviation (n = 3). *p < 0.05, ***p < 0.001 (Student's t-test). (2) The intracellular uptake of DOX by U87-MG cells after crossing BBB by flow cytometry.

The BBB targeting and penetrating efficacy of the dual-targeting dendrimers was investigated in an *in vitro* BBB model based on the transwell inserts cultured with a compact HBMEC monolayer as was reported before (**Figure 6a**) 47. The integrity of the HBMEC cell monolayer was evaluates by the TEER value and the leakage ability of the monolayer. Different DOX formulation were added into the transwell inserts at a DOX concentration of 20 μM when the TEER value of the BBB model was above 250 Ω/cm^2^ and when no leakage was observed within several hours. DOX transported across the* in vitro* BBB model was quantified to evaluate the ability of different DOX formulations to cross the BBB. We found that only ~3.89% of the free DOX was transported through the BBB, while the transported DOX significantly increased after being encapsulated into the dendrimers (transport ratio of ~6.57% for P4PD, ~7.49% for P4PED, and ~10.25% for P4PAD). What's more, about ~11.32% of DOX encapsulated in the dual-targeting dendrimers that were functionalized with both Ang2 and EP-1 crossed the BBB (**Figure 6c**). These results indicated that the dual-functionalization of the dendrimers with the peptides significantly improved the BBB penetrability of the therapeutics. They also demonstrated that Ang2 peptide played a key role in the BBB penetrating by receptor-mediated transcytosis, as was reported in the previous studies 38. The effect of EP-1 peptide to enhance BBB penetration might be due to the synergistic effect by adsorption-mediated transcytosis 44, 45. The enhanced BBB penetration of DOX encapsulated in the non-functionalized dendrimer might be due to the slightly positive charge on the dendrimer that could improve the adsorption-mediate transcytosis.

We further evaluated the dual-targeting effect of the dendrimers in the U87-MG and HBMEC co-culture model. U87-MG cells were seeded into the 12-well plate and were cultured for 24 h, then the established BBB model was put onto the U87-MG cells and different DOX formulations were added into the BBB model (**Figure 6b**). After incubating for 3 h, the inserts were moved and the plates were divided into two groups. U87-MG cells in one group were collected to detect the intracellular uptake of drugs, while U87-MG cells in the other group were cultured for another 24 h and the cell viability was detected by MTS assay. We found that the viability of U87-MG cells decreased in the DOX-loaded dendrimer groups compared to the free DOX group. U87-MG cells exhibited decreased viability when treated with dendrimers functionalized with peptides compared to the PEGylated dendrimer. The viability of U87-MG cells was the lowest when treated with the dual-targeting dendrimer that were functionalized with both EP-1 and Ang2 (**Figure 6d**), indicating the highest anti-glioma efficacy of the dual-targeting dendrimers compared to the other DOX formulations. These results were consistent with the flow cytometry analysis of the intracellular uptake of DOX by U87-MG cells which showed that the intracellular uptake of DOX by the tumor cells was higher in the dendrimer groups compared to free DOX group, and was higher when the cells were treated with peptide-functionalized dendrimers compared to the PEGylated dendrimer. The dual-targeting dendrimers that were functionalized with both EP-1 and Ang2 exhibited the highest intracellular uptake of DOX in U87-MG cells (**Figure 6e**). These results, taken together with the transport ratio of the DOX formulations across the BBB model, demonstrated that the modification of the dendrimers with Ang2 and EP-1 peptides enhanced the BBB penetrability and the glioma targeting efficacy synergistically. The possible mechanism of this dual-targeting effect was speculated as follows: 1) the modification of EP-1 and Ang2 peptides on dendrimer improved its targeting and accumulating effect to BBB, then the dendrimer-based carrier transported across the BBB by Ang2-LRP1 mediated transcytosis and the adsorption-mediated transcytosis. 2) the dendrimers transported across the BBB would target glioma cells via EP-1 and Ang2-incuded targeting effect due to the high expression of EGFR and the moderate expression LRP1 on U87-MG cells.

### 3.6 *In vivo* evaluation of glioma targeting and anti-tumor efficacy

**Figure 7:**
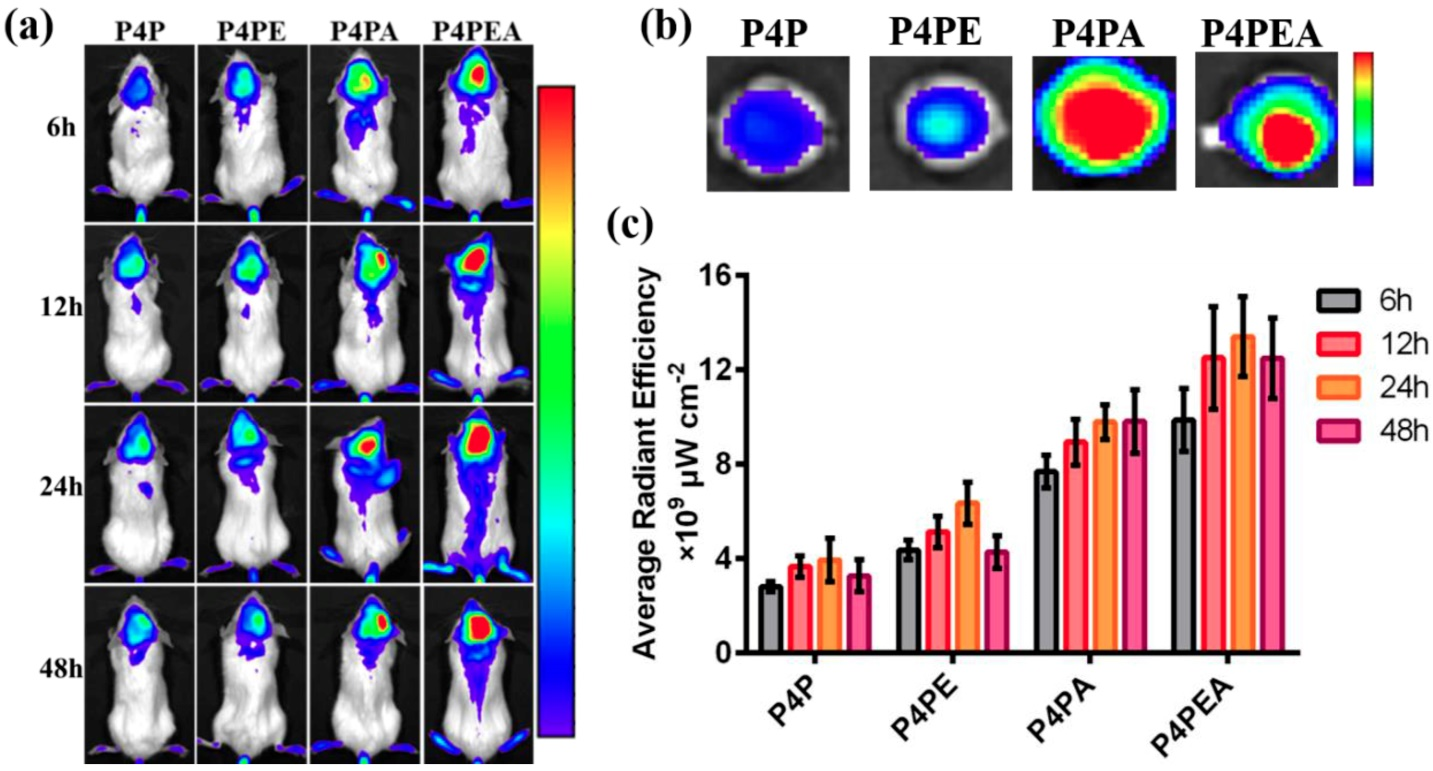
Dual-targeting efficient of the dual-functionalized dendrimer evaluated *in vivo* using glioma-bearing CB-17 SCID mice as model. (a) Dual-targeting effect detected by *in vivo* imaging system after accumulating for 6 h, 12 h, 24 h and 48 h. The glioma-bearing mice were administrated with Cy5.5-labeled dendrimers at a Cy5.5 dose of 2 mg/kg. (b) Glioma-targeting effect of the dual-targeting dendrimer through observing the accumulated Cy5.5-labeled dendrimer in the excised brains at 24 h after administrating. (c) Dual-targeting efficacy was evaluated by calculating the fluorescent intensity accumulated in the brain of the glioma-bearing mice by LiveImaging software.

We first investigated the BBB penetrability and glioma targeting effect of the dual-targeting dendrimer-based carrier using glioma-bearing CB-17 SCID mice *in vivo*. The glioma-bearing CB-17 SCID mice model was established by stereotaxically injecting U87-MG cells into the striatum. The dendrimer-based carriers were labeled by Cy5.5 before administration. Then the labeled dendrimers were injected into the gliomas bearing mice with a Cy5.5 dose of 2 mg/kg *via* tail vein. After accumulating for 6 h, 12 h, 24 h and 48 h, the amount of the Cy5.5 labeled dendrimers which crossed the BBB and accumulated in gliomas was measured using *in vivo* fluorescent imaging system (**Figure 7**). We found that modification of the dendrimers with Ang2 peptide significantly improved the BBB penetrability of the dendrimer-based carriers by LRP1-mediated transcytosis. The dendrimer mono-modified by Ang2 peptide could also target glioma site after crossing the BBB due to the moderated expression of LRP1 on U87-MG cells. As expected, the dual-functionalized dendrimer exhibited the highest fluorescent intensity in the glioma site than the mono-functionalized and PEGylated dendrimer within 48 h, indicating the enhanced BBB penetrating and glioma targeting effect of the dendrimer mediated by Ang2 and EP-1 peptides. The higher fluorescent intensity of EP-1 modified dendrimer than PEGylated dendrimer revealed that EP-1 peptide could improve BBB penetrability by recognizing endothelial cells of BBB and adsorption-mediated transcytosis. The significant enhancement of BBB penetrability and glioma targeting effect demonstrated that the EP-1 and Ang2 peptides played a synergistic effect in the process of glioma targeting. In addition, the biodistribution was also administrated by *in vivo* imaging, the highest fluorescent intensity in kidneys indicated that the major metabolic pathway of drug vehicles was renal metabolism (**Figure S11**).

**Figure 8:**
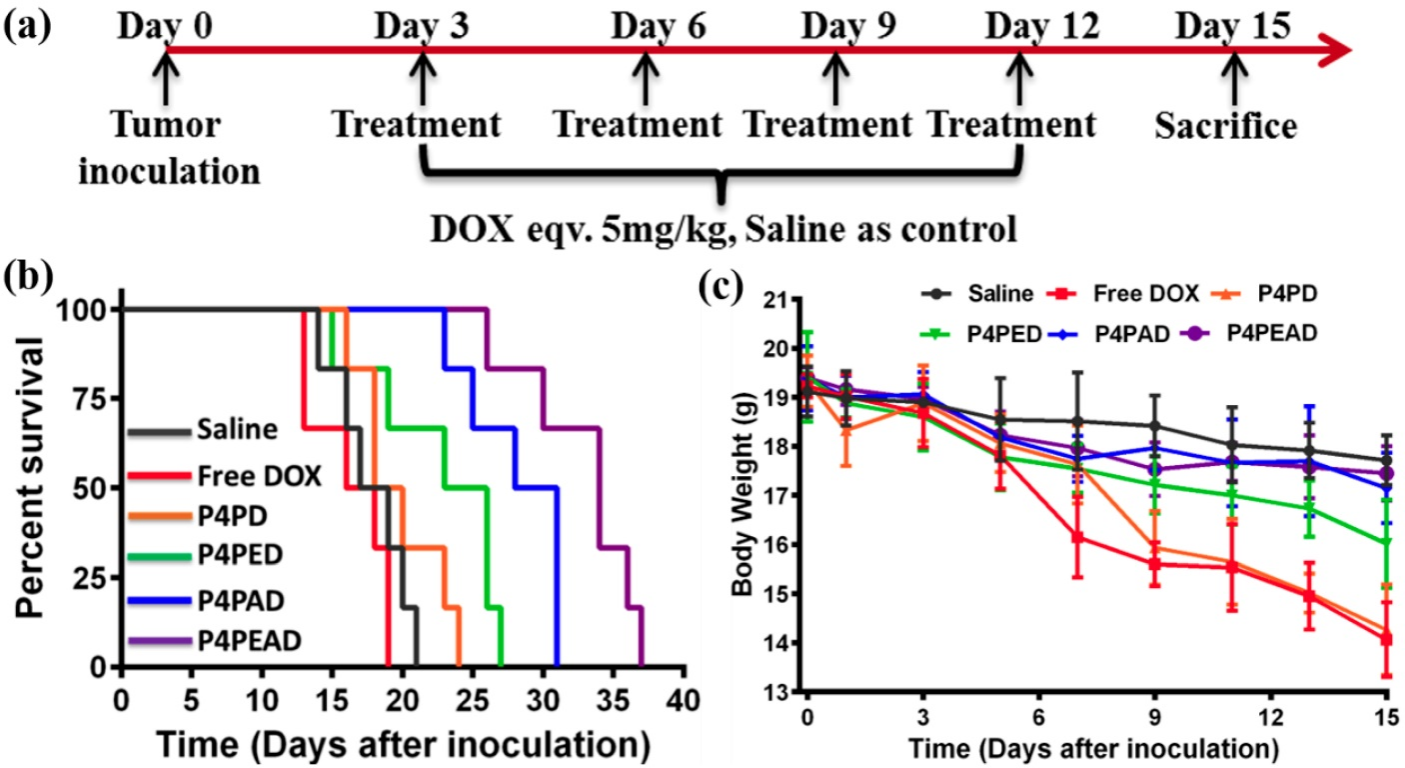
*In vivo* evaluation of the anti-glioma efficacy and the systemic toxicity of the dual-targeting dendrimer. (a) Treatment procedure of the glioma bearing BALB/c nude mice (n = 6). (b) Kaplan-Meier survival curves of different treatment. The Data was recorded after the glioma inoculation. (c) Body weight of the mice during treatment. Error bars represent standard deviation (n = 3).

Furthermore, we investigated the antitumor effect according to the treatment process in gliomas-bearing BALB/c nude mice (**Figure 8a**). We found that the group treated with dual-targeting dendrimer (P4PEAD) exhibited the longest survival time than any other groups, which indicated that the dual-functionalization of the dendrimer remarkably improve the BBB penetrating and glioma targeting effect of the drug delivery system (**Figure 8b**). The controllable drug release in the tumor microenvironment enhanced the anti-tumor effect of the drugs. Meanwhile, the overall survival time was 31 days for Ang2 modified dendrimer (P4PAD) and 27 days for EP-1 modified dendrimer (P4PED), which longer than the group treat with PEGylated dendrimer (PEPD, 24 days) and free DOX (19 days) (**Figure 8b**). These results demonstrated that the peptides modification synergistically improved the anti-glioma effect by improving BBB penetrability and glioma targeting.

We further evaluated the toxicity and side effects of the drug delivery systems. As shown in **Figure 8c**, slight bodyweight loss was observed in the saline treated mice during the entire treatment period. In contrast, the bodyweight of the mice treated with free DOX decreased dramatically after treatment. As expected, the toxicity of DOX was significantly inhibited after being encapsulated into the dual-functionalized and mono-functionalized dendrimers compared with the PEGylated dendrimer and free DOX. That was because the enhanced BBB penetrating and glioma targeting effect of the carriers reduced the concentration of drugs in the circulation and the strong interaction between drugs and carriers in the physiological environment limited the drug leakage. The lack of BBB penetrability of the PEGylated dendrimer and free DOX improved the toxic to normal tissues. As DOX is well known for its cardiotoxicity 64, we performed histological examination of the heart tissues using hematoxylin-eosin (H&E) staining method (**Figure S12**). For the mice administrated with free DOX, the obviously myocardial damage was observed, while there were no obvious damages to the hearts after encapsulating DOX into the carriers. Meanwhile, no obviously damages were observed in the other major organs. In summary, the results confirmed that the dual-targeting carrier effectively improved therapeutic effect of anticancer drugs for glioma while reducing its systemic toxicity *via* the enhanced BBB penetrating ang glioma targeting effect mediated by EP-1 and Ang2 peptides.

## 4. Conclusion

In this work, we obtained an EGFR-targeting peptide-1 (EP-1) by peptide screening, and demonstrated the high affinity and specificity of this peptide at both molecular and cellular levels. Then we developed a dual-targeting drug delivery system based on the fourth generation PAMAM dendrimer conjugated with EP-1 and Ang2, a peptide that could induce BBB transport through binding to LRP1 that was highly expressed in the endothelial cells of BBB. DOX was encapsulated into the interior cavities of the dendrimers. This dual-functionalized carrier could release the anticancer drugs responding to the weak acidic microenvironment of tumor. Additionally, the dual-targeting dendrimer-based carriers exhibited enhanced BBB penetrability and glioma targeting effect both *in vitro* and *in vivo* due to the synergistic effect of the two peptides. We further demonstrated that the dual-targeting drug delivery system significantly enhanced the therapeutic efficacy of DOX for glioma and reduced the systemic toxicity of DOX *in vivo* through the enhanced BBB penetrating and glioma targeting of the combined peptide ligands. This smart dendrimer-based carrier not only demonstrated a promising strategy in glioma therapy, but also showed a strategy to overcome the BBB through peptide stapling technique.

## Supplementary Material

Supplementary figures and tables.

## Abbreviations

Ang2: Angiopep-2 peptide
EP-1: EGFR-targeting peptide-1
PAMAM: ploy(amidoamine) dendrimer
P4: Generation fourth PAMAM
P4P: PEGylated generation fourth PAMAM
P4PE: EP-1 modified P4P
P4PA: Ang2 modified P4P
P4PEA: EP-1 and Ang2 co-modified P4P
P4PD: DOX-loaded P4P
P4PED: DOX-loaded P4PE
P4PAD: DOX-loaded P4PA
P4PEAD: DOX-loaded P4PEA
