# Supplementary material for: Enhanced blood-brain-barrier penetrability and tumor-targeting efficiency by peptide-functionalized poly(amidoamine) dendrimer for the therapy of gliomas
Source: Nanotheranostics. 2019 Sep 19;3(4):311–30. doi: 10.7150/ntno.38954 (PMC6821994; doi:10.7150/ntno.38954)
Supplement: Supplementary file 1 — Supplementary figures and tables. [file ntnov03p0311s1.pdf]

Supporting Information

Enhanced blood-brain-barrier penetrability and tumor-targeting efficiency by peptide-functionalized dendrimer for the therapy of gliomas

Changliang Liu[a, b, #], Zijian Zhao[a, #], Houqian Gao[a, b], Iman Rostami[a], Qing You[a, b], Xinru Jia[c], Chen Wang[a, b, *], Ling Zhu[a, *], Yanlian Yang[a, b, *]

[a] CAS Key Laboratory of Standardization and Measurement for Nanotechnology

CAS Key Laboratory of Biological Effects of Nanomaterials and Nanosafety

CAS Center for Excellence in Nanoscience

National Center for Nanoscience and Technology, Beijing 100190, China

[b] University of Chinese Academy of Sciences, Beijing 100049, China

[c] Department of Chemistry, Peking University, Beijing 100871, China

E-mail: wangch@nanoctr.cn, zhul@nanoctr.cn, yangyl@nanoctr.cn

[#] These authors contributed equally to this paper.

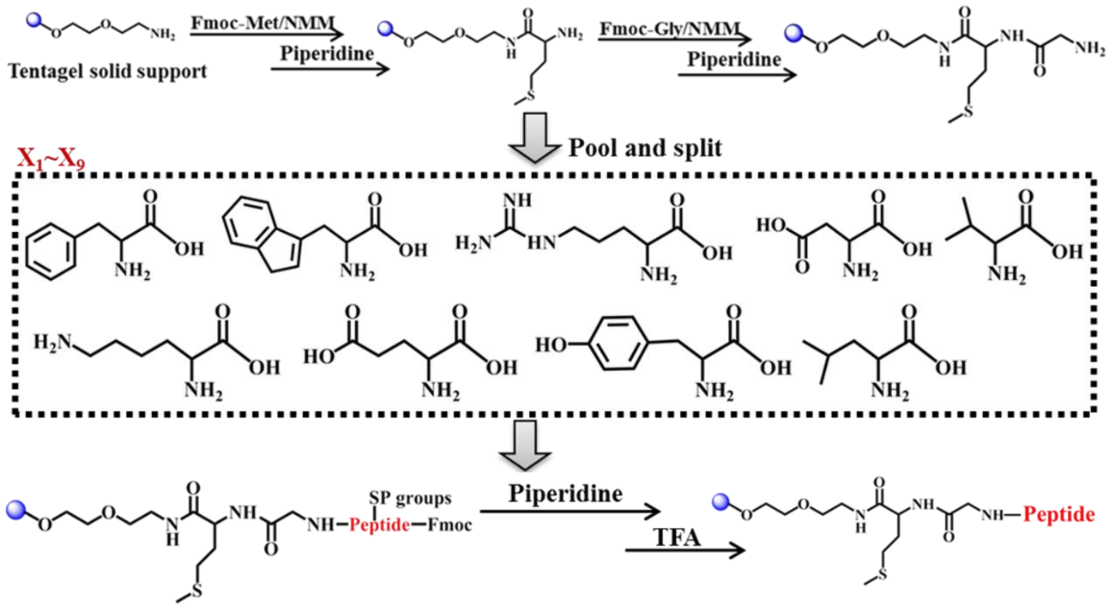

Scheme S1. Construction and synthesis of the OBOC peptide library towards EGFR.

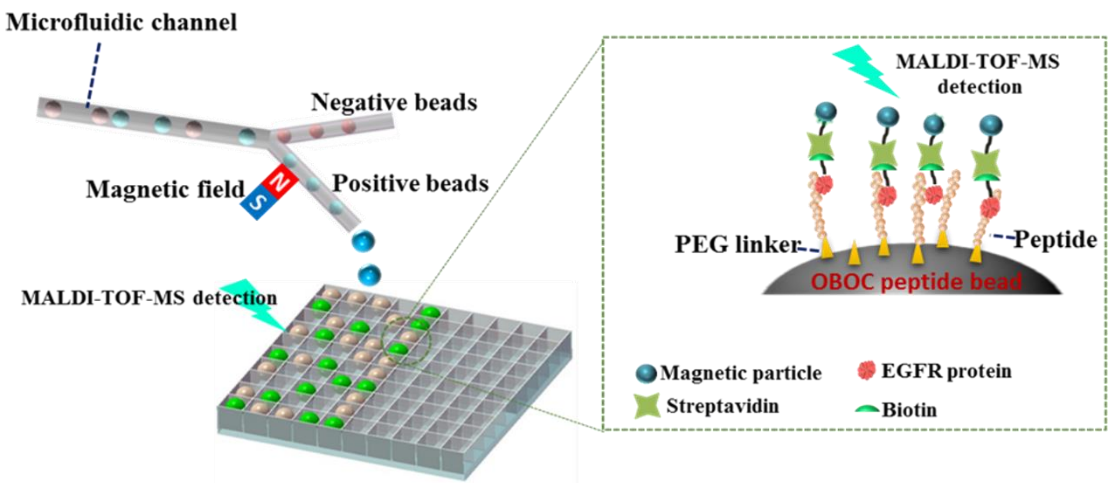

Scheme S2. Schematic illustration of trapping and sorting of the positive peptide beads through microchannel in the presence of magnetic field.

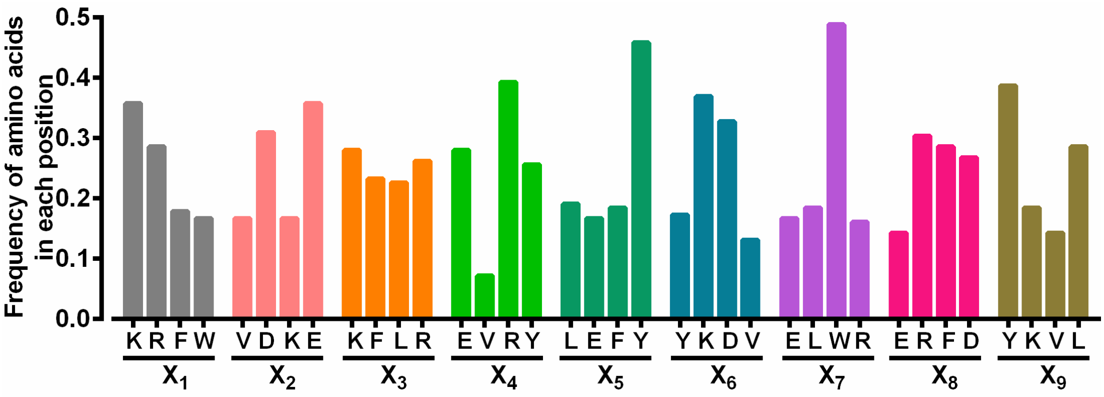

Figure S1. Frequency of amino acids in each position of noncanonical EGFR binding peptides detected by sequence alignment using the ClustalX2 multiple alignment tool.

Peptide	Sequence	Molecular weight
**EP-1**	**KERRYKWDYGC**	**1503.7**
**EP-2**	**KDKEYDWRYGC**	**1462.6**
**EP-3**	**KDLYYKWFYGC**	**1485.7**
**EP-4**	**KEFRYKWFLGC**	**1483.8**
**EP-5**	**REKEYKWDYGC**	**1504.6**

Table S1. Peptide sequences redesigned according to the alignment.

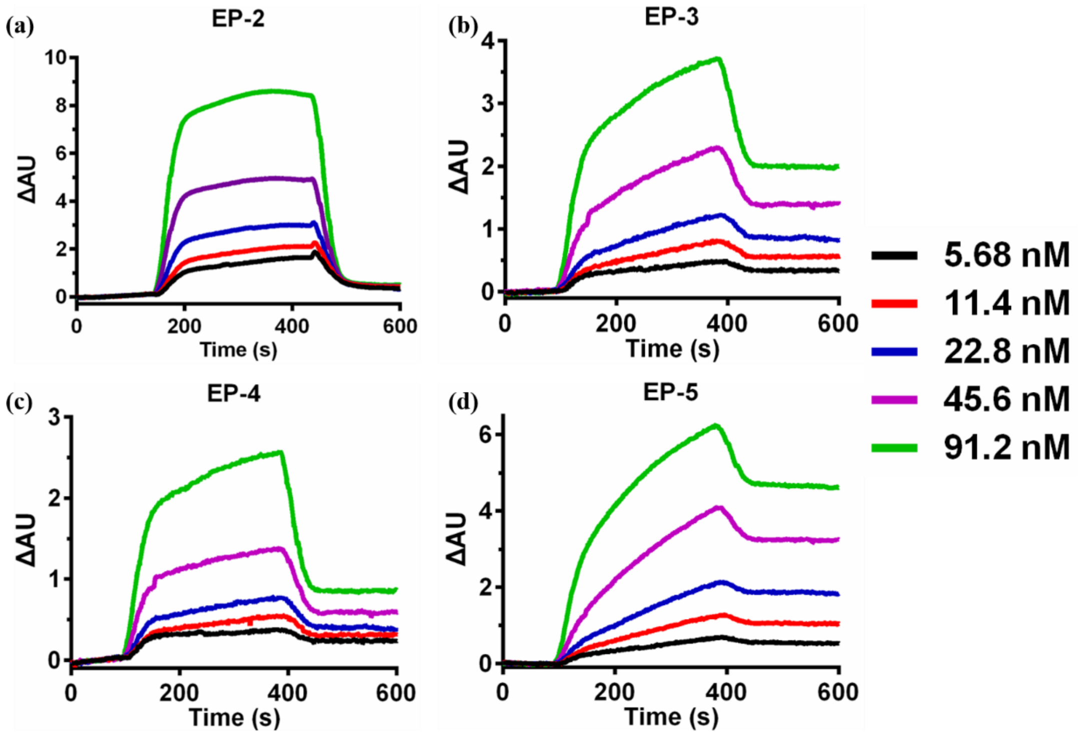

Figure S2. Evaluation of the affinity and specificity of (a) EP-2, (b) EP-3, (c) EP-4 and (d) EP-5 peptide towards EGFR by surface plasmon resonance imaging (SPRi).

Peptide	k_a (1/mol L^{-1} s)	k_d (1/s)	K_D (mol/L)
**EP-1**	8.12×10^{4}	1.23×10^{-5}	1.51×10^{-9}
**EP-2**	593	5.32×10^{-4}	8.97×10^{-7}
**EP-3**	6.69×10^{4}	6.47×10^{-5}	9.66×10^{-10}
**EP-4**	9×10^{4}	2.58×10^{-5}	2.87×10^{-10}
**EP-5**	9.98×10^{4}	1.22×10^{-4}	2.81×10^{-9}

Table S2. The equilibrium dissociation constant (K_D) values between the peptides and EGFR were fitted using BIAevaluation version 4.1 software (Biacore, Inc.) according to the SPRi sensorgram.

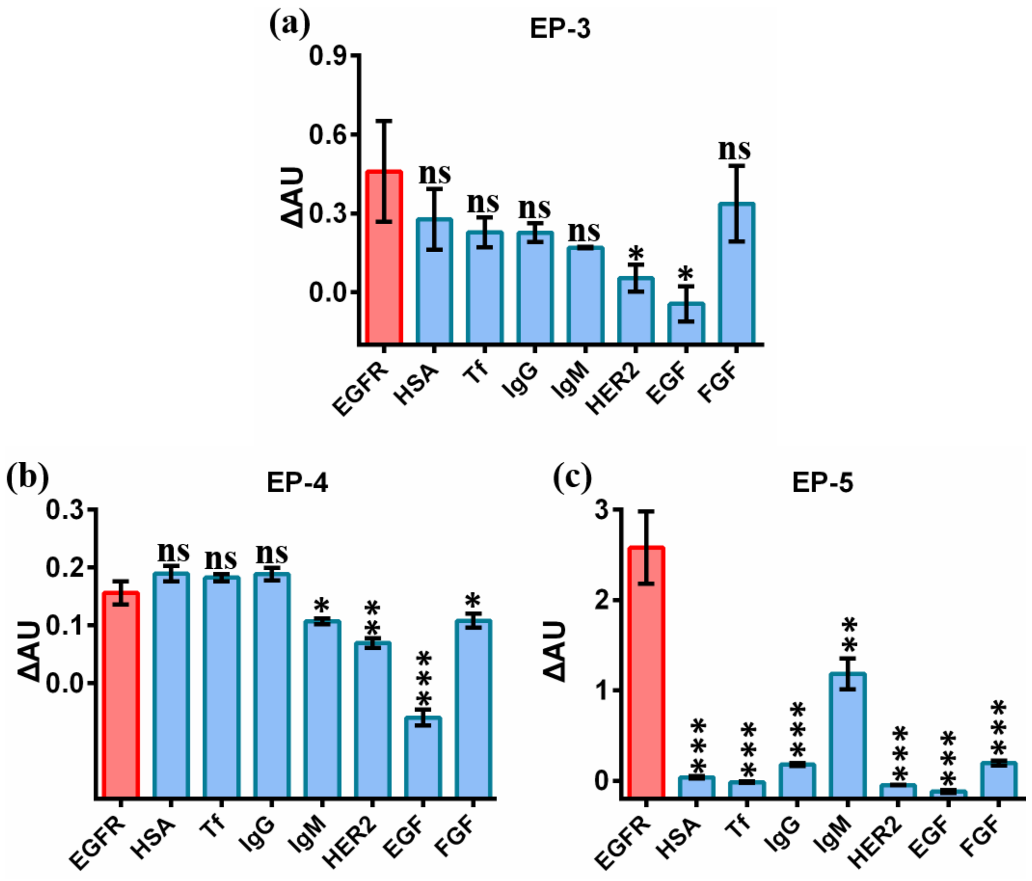

Figure S3. SPRi binding signals of (a) EP-3, (b) EP-4 and (c) EP-5 towards EGFR, HSA, Tf, IgG, IgM, HER2, EGF and FGF. Error bars represent the standard deviation (n = 3). *p < 0.05, **p < 0.01, ***p < 0.001, as compare with EGFR.

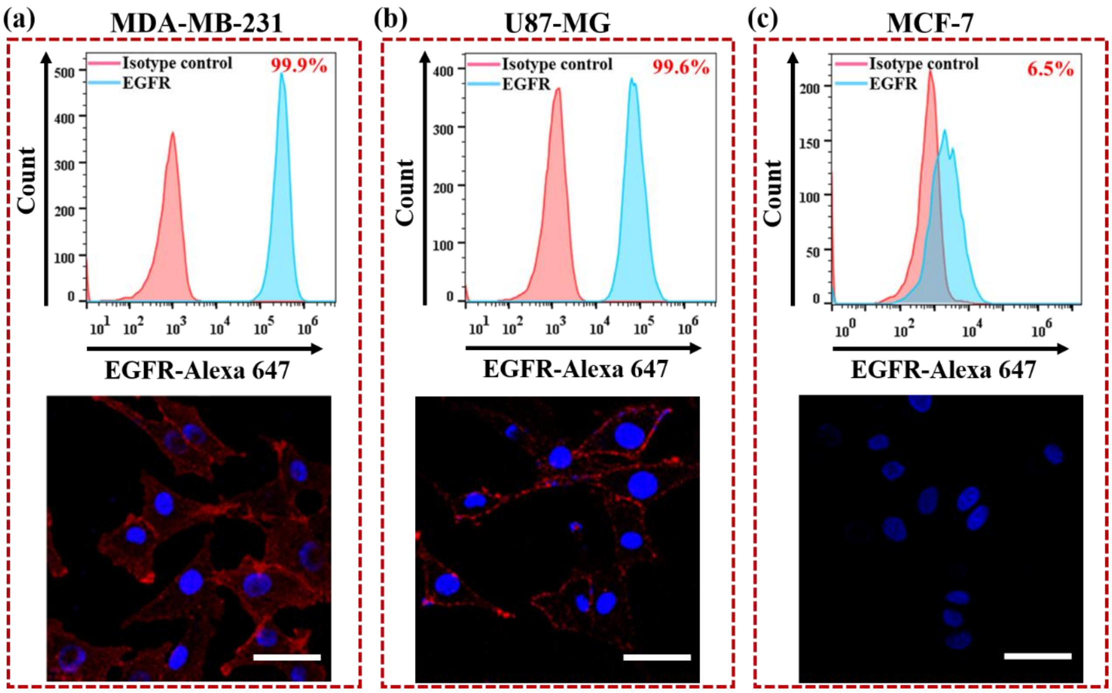

Figure S4. Evaluation of the expression of EGFR in (a) MDA-MB-231, (b) U87-MG and (c) MCF-7 cells by flow cytometry (up) and confocal imaging (bottom). For flow cytometry, the cells were labeled with anti-EGFR-Alexa 647. Isotype IgG was used as negative control. For confocal microscopic imaging, the cells were labeled with anti-EGFR-Alexa 647 and the nuclei stained by DAPI. Scale bar: 50 μm.

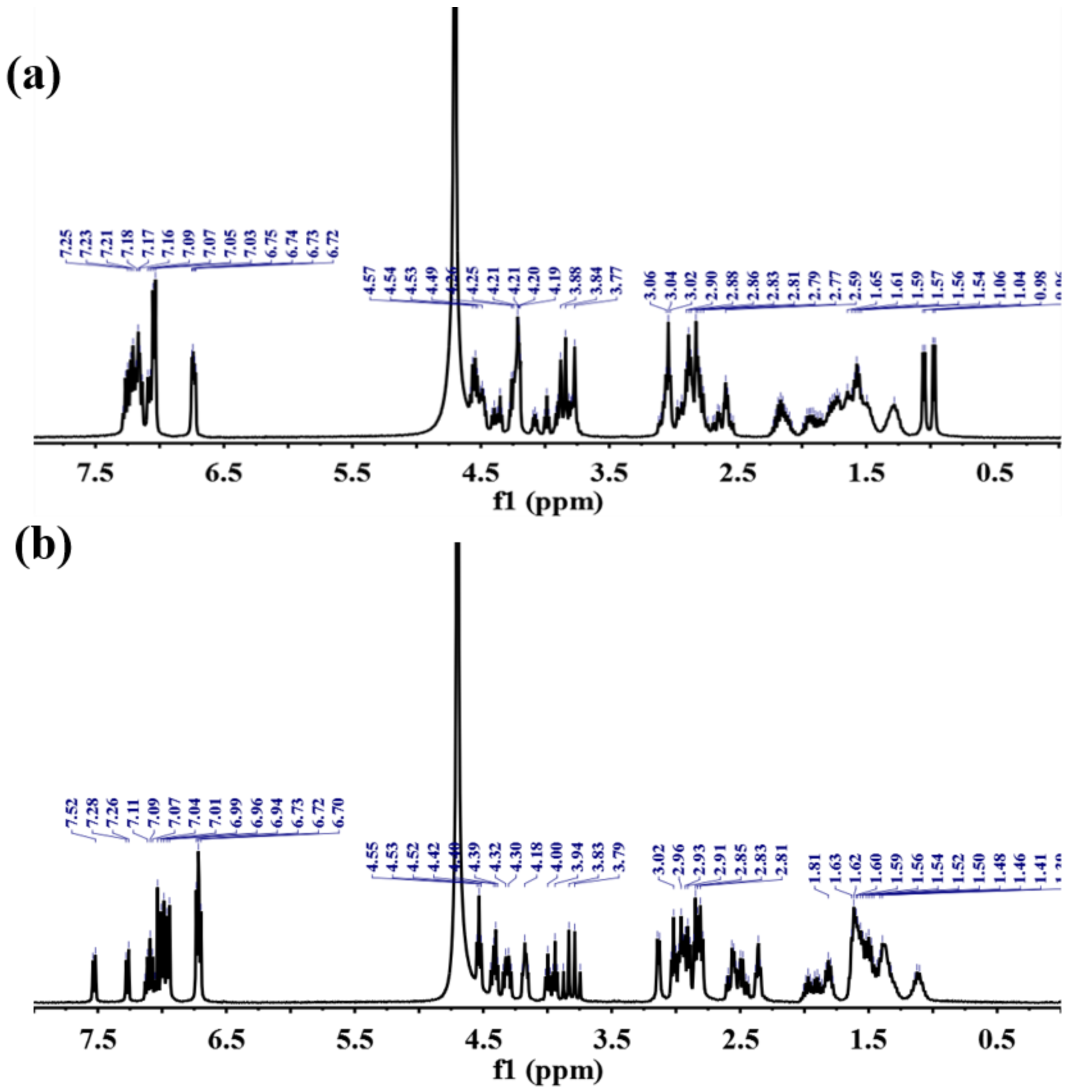

Figure S5. ^{1}H NMR spectrum of (a) Ang2 and (b) EP-1 peptide in D_2O.

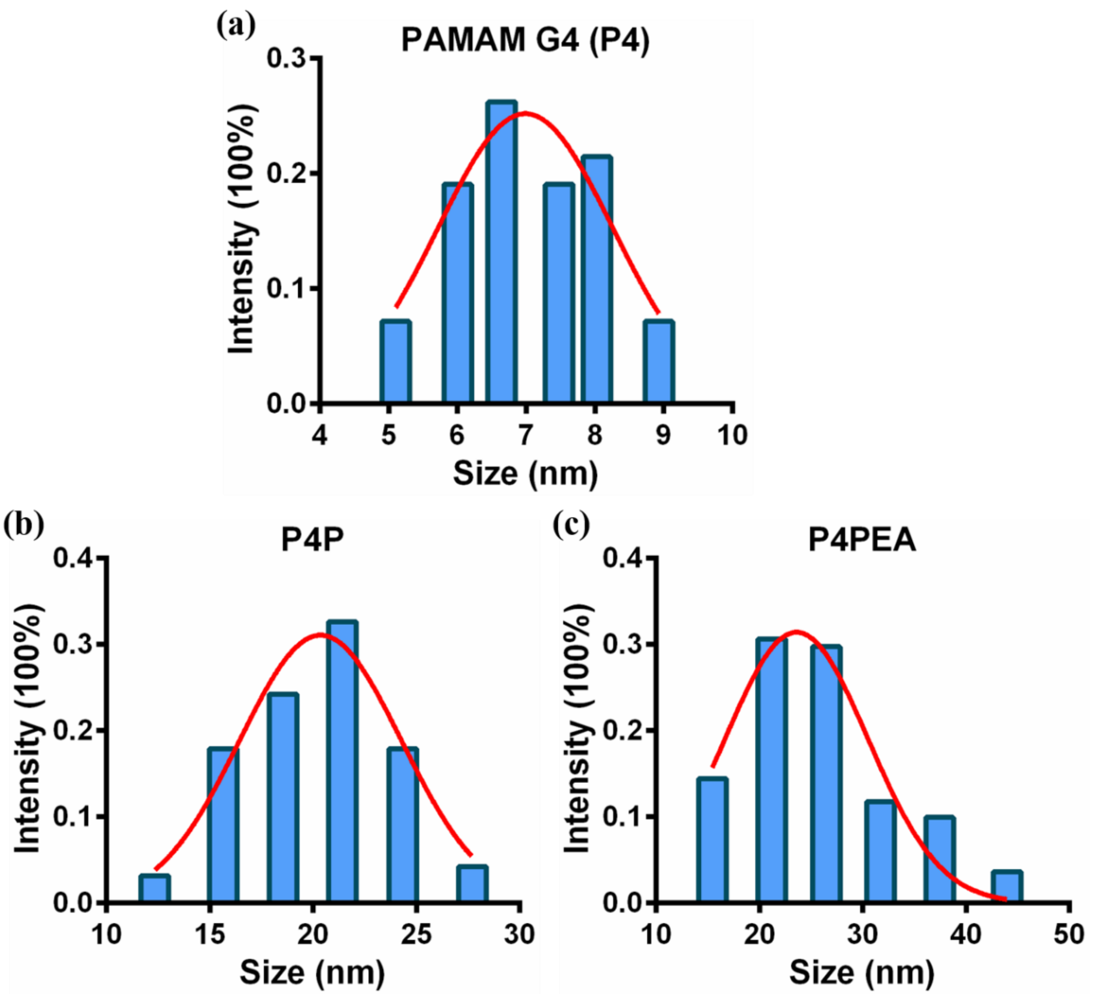

Figure S6. Size distribution of the dendrimer-based carriers determined by analyzing TEM images using Nanomeasure 1.2.0 software.

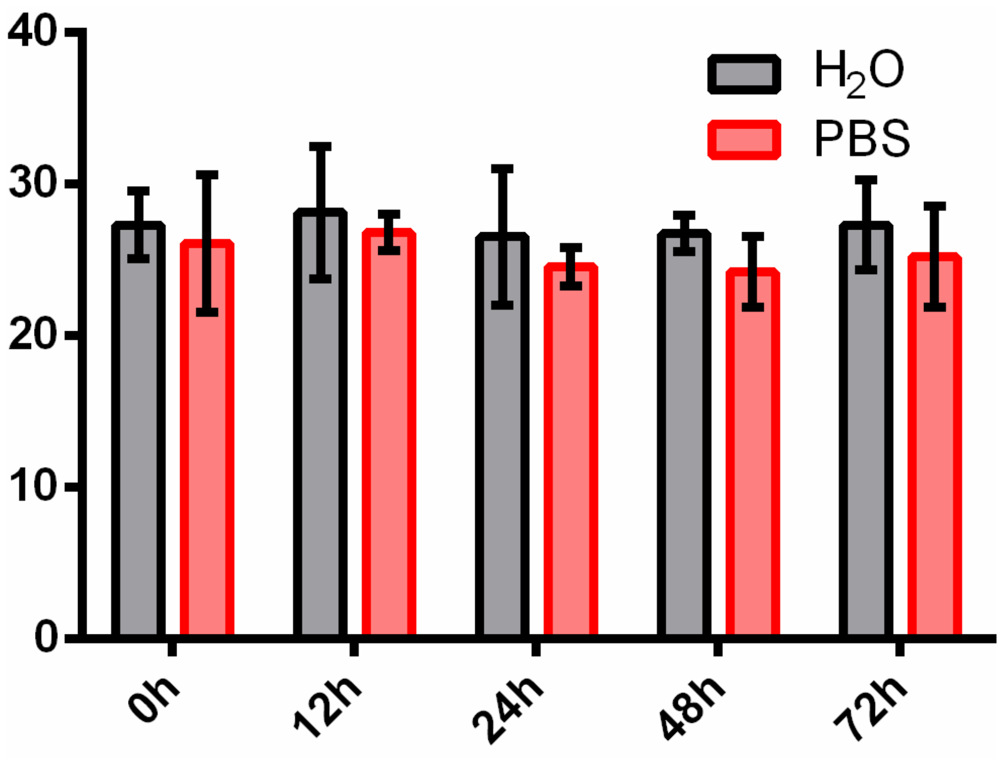

Figure S7. Stability test of the dual-functional dendrimer-based carrier by DLS in H_2O and PBS buffer.

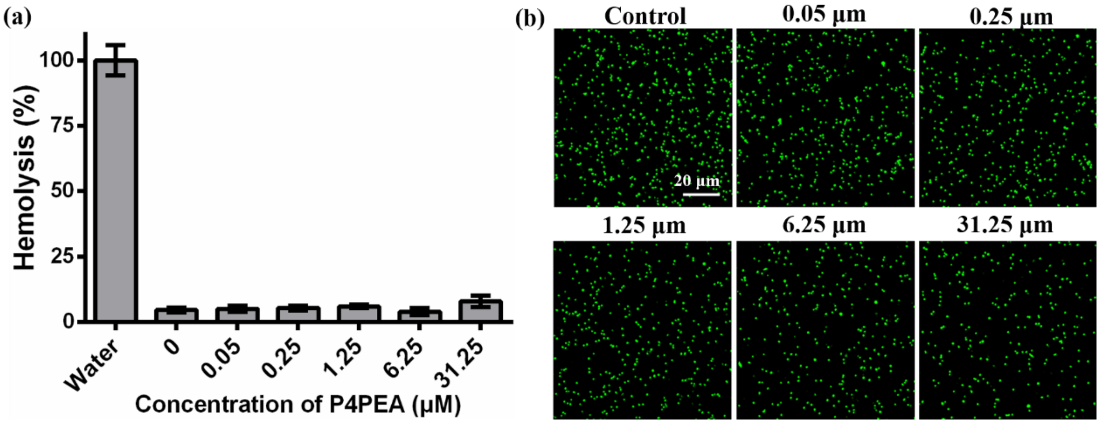

Figure S8. (a) Hemolysis quantification of RBCs at various concentrations of P4PEA. (b) Cell viability of HBMEC cells after being treated with the dual-functional dendrimer for 24 h determined by the live/dead assay.

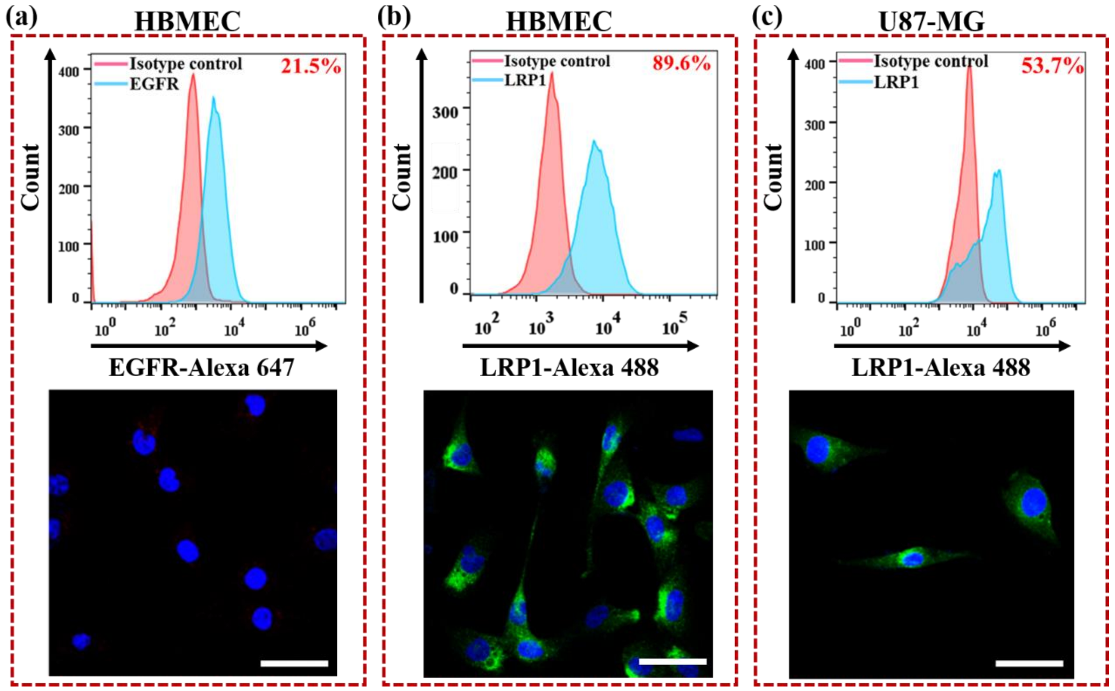

Figure S9. (a) Evaluation of the expression of EGFR in HBMEC cells by flow cytometry (up) and confocal imaging (bottom). The cells were labeled with anti-EGFR-Alexa 647. Isotype IgG was used as negative control. Scale bar: 50 µm. (b, c) Evaluation of the expression of LRP1 in (b) HBMEC cells and (c) U87-MG cells by flow cytometry (up) and confocal imaging (bottom). The cells were labeled with anti-LRP1-Alexa 488. Isotype IgG was used as negative control. Scale bar: 50 µm.

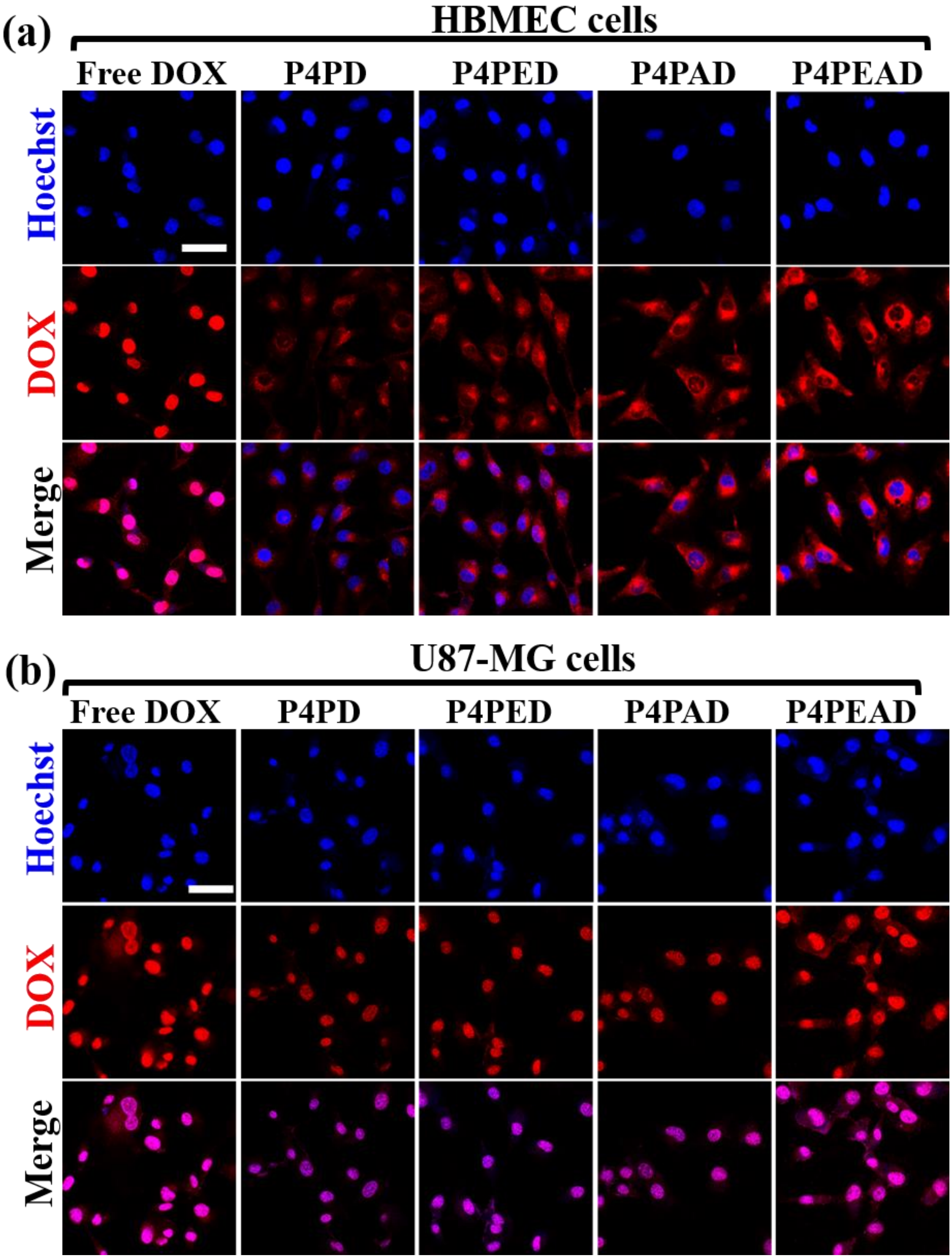

Figure S10. Intracellular uptake of different DOX formulations (Free DOX, P4PD, P4PED, P4PAD and P4PEAD) by (a) HBMEC and (b) U87-MG cells detected by laser scanning confocal microscope (LSCM). Cells were incubated with different DOX formulations for 2 h at the DOX concentration of 20 μM. Scare bare: 50 μm.

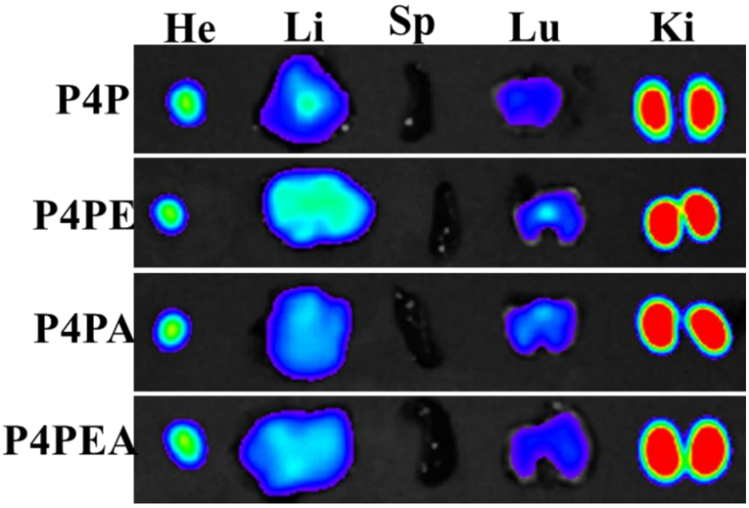

Figure S11. Bio-distribution of Cy5.5-labeled P4P, P4PE, P4PA and P4PEA in major organs of tumor-bearing nude mice photographed at 24 h.

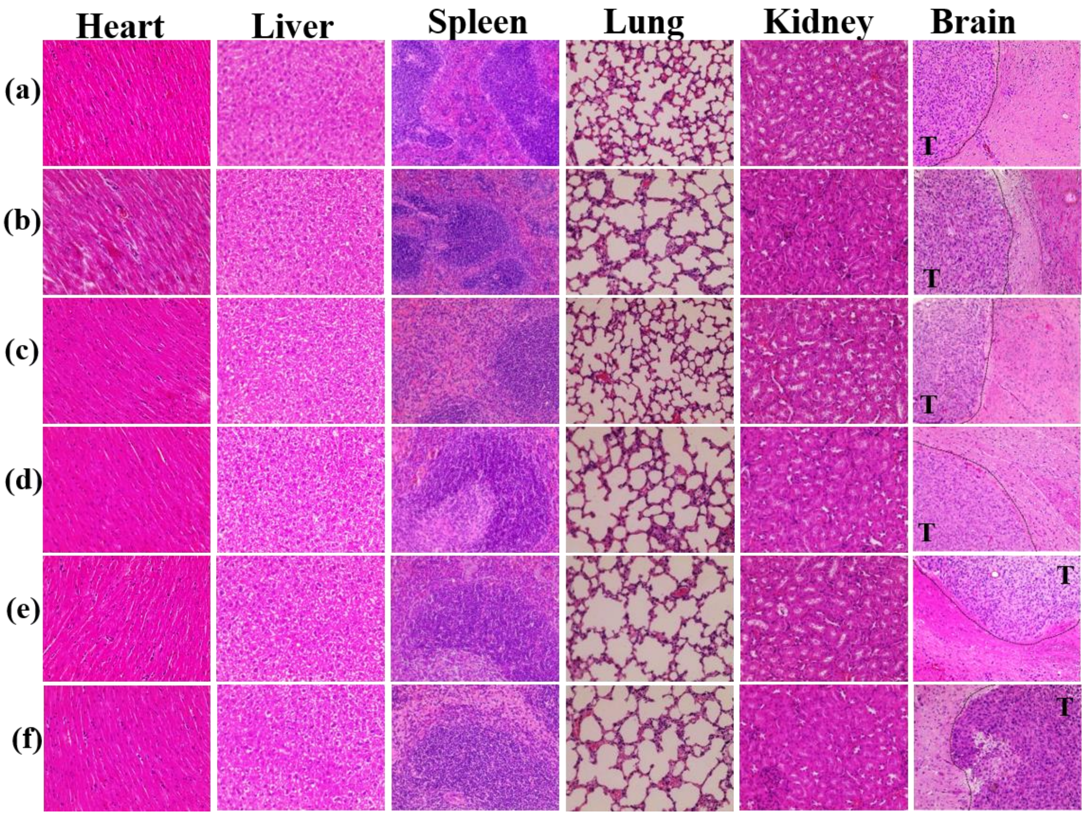

Figure S12. Histological analysis of the major organs of the glioma-bearing mice after being treated with different DOX formulations with 5mg/kg equivalent dose of DOX, (a) Saline, (b) free DOX, (c) P4PD, (d) P4PED, (e) P4PAD and (f) P4PEAD. H&E results exhibited the inhibited cardiotoxicity of DOX by dendrimer-based carriers. Tumor tissue has been marked by “T” in the brain images.
